# Phosphodiester Silybin Dimers Powerful Radical Scavengers: A Antiproliferative Activity on Different Cancer Cell Lines

**DOI:** 10.3390/molecules27051702

**Published:** 2022-03-05

**Authors:** Valeria Romanucci, Rita Pagano, Antonio Lembo, Domenica Capasso, Sonia Di Gaetano, Armando Zarrelli, Giovanni Di Fabio

**Affiliations:** 1 Department of Chemical Sciences, University of Naples Federico II, Via Cintia 4, 80126 Napoli, Italy; valeria.romanucci@unina.it (V.R.); rita.pagano@unina.it (R.P.); antonio.lembo@unina.it (A.L.); zarrelli@unina.it (A.Z.); 2 Interuniversity Research Centre on Bioactive Peptides (CIRPeB), University of Naples “Federico II“, Via Mezzocannone 16, 80134 Napoli, Italy; domenica.capasso@unina.it (D.C.); digaetan@unina.it (S.D.G.); 3 Center for Life Sciences and Technologies (CESTEV), University of Naples “Federico II“, Via De Amicis 95, 80145 Napoli, Italy; 4 Institute of Biostructures and Bioimaging-CNR, Via Mezzocannone 16, 80134 Napoli, Italy; 5 AIPRAS Onlus (Associazione Italiana per la Promozione delle Ricerche sull’Ambiente e la Salute umana Onlus), Via Campellone 50, 82030 Dugenta, Italy

**Keywords:** silybin, silibinin, flavonolignan dimers, radical scavenger of ROS, apoptosis, leukemia cells

## Abstract

Silibinin is the main biologically active component of silymarin extract and consists of a mixture 1:1 of two diastereoisomeric flavonolignans, namely silybin A (**1a**) and silybin B (**1b**), which we call here silybins. Despite the high interest in the activity of this flavonolignan, there are still few studies that give due attention to the role of its stereochemistry and, there is still today a strong need to investigate in this area. In this regard, here we report a study concerning the radical scavenger ability and the antiproliferative activity on different cell lines, both of silybins and phosphodiester-linked silybin dimers. An efficient synthetic strategy to obtain silybin dimers in an optical pure form (**6aa**, **6ab** and **6bb**) starting from a suitable building block of silybin A and silybin B, obtained by us from natural extract silibinin, was proposed. New dimers show strong antioxidant properties, determined through hydroxyl radical (HO^●^) scavenging ability, comparable to the value reported for known potent antioxidants such as quercetin. A preliminary screening was performed by treating cells with 10 and 50 μM concentrations for 48 h to identify the most sensitive cell lines. The results show that silibinin compounds were active on Jurkat, A375, WM266, and HeLa, but at the tested concentrations, they did not interfere with the growth of PANC, MCF-7, HDF or U87. In particular, both monomers (**1a** and **1b**) and dimers (**6aa**, **6ab** and **6bb**) present selective anti-proliferative activity towards leukemia cells in the mid-micromolar range and are poorly active on normal cells. They exhibit different mechanisms of action in fact all the cells treated with the **1a** and **1b** go completely into apoptosis, whereas only part of the cells treated with **6aa** and **6ab** were found to be in apoptosis.

## 1. Introduction

Oxidative stress can cause cell injury and death, which may be related to numerous diseases and conditions, such as liver damage, aging, cancer, stroke, Alzheimer’s disease, and Parkinson’s disease [1,2]. The well-known capability of flavonoids to scavenge reactive oxygen species (ROS) is frequently cited as the key property underlying the prevention of and/or reduction in oxidative stress-related chronic diseases and age-related disorders, such as cardiovascular diseases, carcinogenesis, and neurodegeneration. However, many studies have suggested that the therapeutic activity of these compounds involves other properties their ability to directly bind to target peptides [3], inducing the inhibition of key enzymes, the modulation of cell receptors or transcription factors, as well as the perturbation of protein (or peptide) aggregates, who are known to regulate many cell functions.

**Figure 1:**
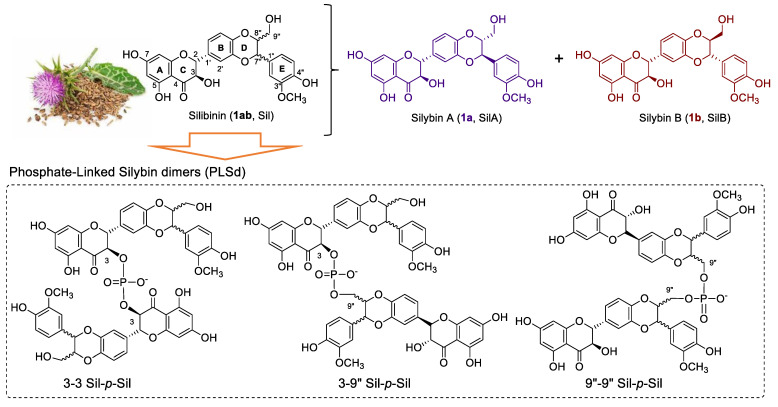
Structure of silibinin (**1ab**), silybin A (**1a**), silybin B (**1b**) and Phosphate-Linked Silybin dimers (PLSd) [8].

Flavonoids have a broad spectrum of biological activities and administering high dosages could trigger side effects. One strategy to improve the potency and selectivity of flavonoids is to take advantage of the dimeric nature of biflavonoids, thereby facilitating simultaneous interactions through the binding of multiple sites of a biological target [4,5,6]. Similar to flavonoid dimers, flavonolignan dimers or simply bi-flavonolignans are also an emerging class of dimeric compounds that unlike bi-flavonoids, which are very widespread in nature, consist of synthetic dimers of few flavonolignans isolated from the milk thistle *Silybum marianum* [L. Gaertn. (Asteraceae)] [7]. In this frame, recently we reported the synthesis of new silibinin dimers in which the two monomer units are linked through a phosphodiester bridge, between two aliphatic OH functions (Phosphate-Linked Silybin dimers, Figure 1) [8].

Silibinin is a diastereoisomeric mixture of two flavonolignans, namely, silybin A (SilA) and silybin B (SilB) (**1a** and **1b**, Figure 1), in a ratio of approximately 1:1, extracted from milk thistle seeds [9,10]. Silibinin has been used as a traditional drug to treat a range of liver disorders, including hepatitis and cirrhosis. The manifold inhibitory effects of silibinin against various cancer cells include growth inhibition, anti-inflammation, cell cycle regulation, apoptosis induction, chemo-sensitization, inhibition of angiogenesis, reversal of multi-drug resistance, and inhibition of invasion and metastasis [11,12]. Many in vitro and in vivo reports on the activity of silibinin, clearly neglect the structure-activity relationship of the pair of diastereoisomers, using silibinin, the natural mixture of the two flavonolignans SilA and SilB, for all experiments. These studies have mostly disregarded this aspect because of the difficulty separating, on a preparative scale, two diastereoisomers (**1a** and **1b**). In the 2021 a very in-depth study by Kren et al. [13] on the central role of stereochemistry in the pharmacological properties of silybin, highlights how it is necessary to continue studying these flavonolignans, together with the other silymarin flavonolignans, never neglecting their optical purity.

As a part of our continuing research effort on the synthesis of newly modified silibinin [14,15,16], in 2017, starting from silibinin (**1ab**), dimers 3-3, 3-9′′′ and 9′′-9′′ phosphodiester were obtained (Figure 1) [8]. Dimers, obtained as mixture of diastereoisomers, were very soluble in water and stable in both human serum and alkaline phosphatase. Despite silibinin (**1ab**) and silybins (**1a** and **1b**) [17,18] not having strong antioxidant activity, dimers 9′′-9′′ showed a strong radical-scavenging ability. In particular, the ability to scavenge ^1^O_2_ in H_2_O was tested, and a higher reactivity towards HO^●^ (about two times) was estimated for the 3-9′′ and 9′′-9′′ dimers with respect to silibinin. Starting from these results, it seemed interesting to investigate the structure–activity relationships of dimers 9′′-9′′, obtained from diastereoisomerically pure silybin monomers (SilA **1a** and SilB **1b**, Figure 1).

Herein, we report an improvement of 9′′-9′′ PLSd dimers synthesis and a systematic study on the ability to scavenge HO^●^ radicals as well as their antiproliferative effect on many human tumor cell lines of different histological origins or metastatic potential. Human dermal fibroblasts (HDFs) were used as healthy cells to evaluate the selectivity of action of the examined metabolites towards tumor cells. Furthermore, apoptosis induction was investigated in leukemia cells treated with the examined compounds.

## 2. Results and Discussion

### 2.1. Synthesis of 9′′-9′′ Phosphodiester Silybin Dimers 6

To deepen our research efforts on 9′′-9′′ phosphodiester silybin dimers, we chose to improve the efficiency of the initially proposed synthetic strategy [18,19,20]. For the synthesis of these highly symmetrical dimers, it was advantageous to start from the intermediate 9′′-OH of the silybins; therefore, it was necessary to review the previously reported synthetic strategy [8].

In the previous method, the synthesis involves the regioselective protection of different OH functions of silybin with isobutyryl chloride, but it is laborious and not very efficient (yields ≤ 20%) [7]. To develop an efficient synthetic strategy and highlight the effects of stereochemistry on biological activity, we started from two pure diastereoisomers, silybin A and silybin B (**1a** and **1b**), obtained by our own silibinin purification protocol [21].

**Scheme 1:**
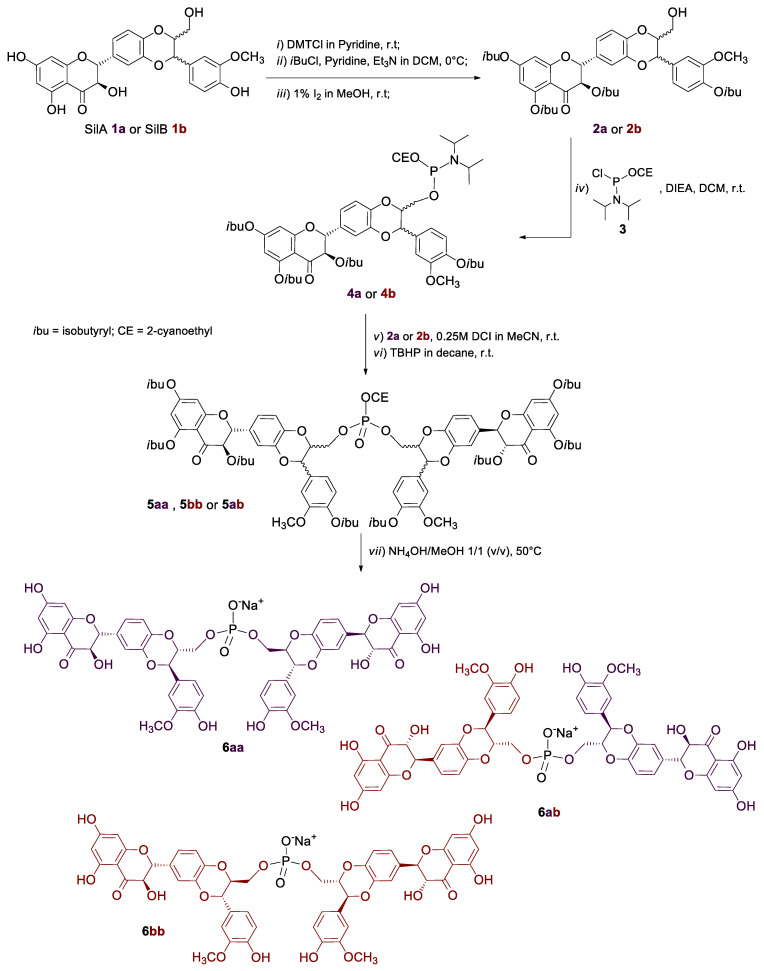
Synthesis of 9′′-9′′ linked-phosphate silybin dimers **6aa**, **6bb** and **6ab**.

Initially, we converted silybins (**1a** and **1b**) into their 9′′-ODMT ether (Scheme 1) and then applied exhaustive acylation with an excess of isobutyryl chloride in DCM and pyridine. Fully protected silybins were obtained in good yields (65–68% range, see Materials and Methods section). The next treatment with 1% I_2_ in MeOH allowed the removal of the DMT protecting group to give **2** (**2a** or **2b**, Scheme 1) in 90% and 88% yields, respectively [22]. Unlike our previously reported observations during formic acid deprotection [19,20,23], side deprotection products were not observed with this procedure. In addition, deprotection was highly reproducible and very efficient. Phosphitylation of building blocks **2a** and **2b** with 2-cyanoethyl-N,N-diisopropylaminochlorophosphoramidite (**3**) in anhydrous DCM led to derivatives **4a** and **4b** in 86% and 80% yields, respectively.

These were then coupled with building blocks **2a** and **2b** in the presence of 4,5-dicyanoimidazole (DCI) in MeCN. For the synthesis of the heterodimer SilA-p-SilB, the best yield was observed by coupling silybin A phosphoramidite **4a** and the protected silybin B (**2b**). After one-pot oxidation of the triester phosphite to phosphate with *t*ButOOH in decane, the phosphotriester dimers were purified and obtained in good yields (**5aa** 83%, **5bb** 80% and **5ab** 77%). Treatment with ac. ammonia finally led to complete deprotection, and after RP-HPLC purification, dimers **6aa**, **6bb** and **6ab** were obtained in 77%, 82% and 80% yield, respectively. The structures of dimers **6** were confirmed by 1D and 2D NMR (^1^H, ^13^C, and ^31^P) and MS analyses. The spectra of the silybin derivatives **2a** and **2b** appeared as those of a silybin monomer (see Experimental Section and Supporting Material), whereas the splitting of some signals is observed in the spectra of phosphoramidites **4a** and **4b**, since they are a pair of diastereoisomers where the phosphorous is a stereocenter and it can be *R* or *S*. The same goes for dimers **5aa** and **5bb** and **5ab**, which are also a mixture of diastereoisomers. As expected, for the **6ab** dimer, chemical shift splitting of some nuclei was observed in both the ^1^H and ^13^C spectra.

### 2.2. Radical Scavenger Activities (HO^●^)

Biologically, the hydroxyl radical (HO^●^) is widely believed to be generated when hydrogen peroxide reacts with Fe(II) (Fenton reaction). The putative HO^●^∙is an extremely reactive and short-lived species that can damage DNA, proteins, and lipids. However, the Fe(II)/H_2_O_2_ mixture has disadvantages in a scavenging assay because many flavonoids as well as flavonolignans are also metal chelators. When the sample is mixed with Fe(II), it may alter the activity of Fe(II) by chelation. As a result, it is impossible to distinguish if the antioxidants are simply good metal chelators or HO^●^ scavengers. In our study the second order rate constants for HO^●^ reactions with silybin dimers (**6aa**, **6bb** and **6ab**) have been determined by pulse photolysis method [24] using hydrogen peroxide (H_2_O_2_) as ROS sources. The reactivity towards HO^●^ was determined to be in the same order of magnitude for dimers **6aa**, **6bb** and **6ab** (Table 1), always remaining significantly greater than dimers 3-3 and 3-9′′ [8]. This could be explained considering that in dimers 9′′-9′′ the 3, 5 and 4′′ OH functions, responsible for the radical scavenger activity, are not involved in any bond. In fact, the influence of the individual hydroxy groups of silibinin (**1ab**) on its antioxidant and radical scavenging properties were studied in detail and the findings led to the conclusion that the 3, 5 and 4′′ phenolic moieties as well as the 3-OH group, are essential for the compounds radical- scavenging properties [25,26].

### 2.3. In Vitro Antiproliferative Activity of Dimers 6aa, 6ab, and 6bb

The anticancer efficacy of silibinin, which is mainly realized through targeting proliferation, apoptosis, inflammation, angiogenesis, and other cancer-modulating mechanisms, is clearly evident from recently published reports. Despite the high interest in the properties of this flavonolignan, which is also attributable to its non-toxicity, pharmacological studies on the two diastereoisomers, silybins A and B, are still required to develop a structure–activity profile.

**Figure 2:**
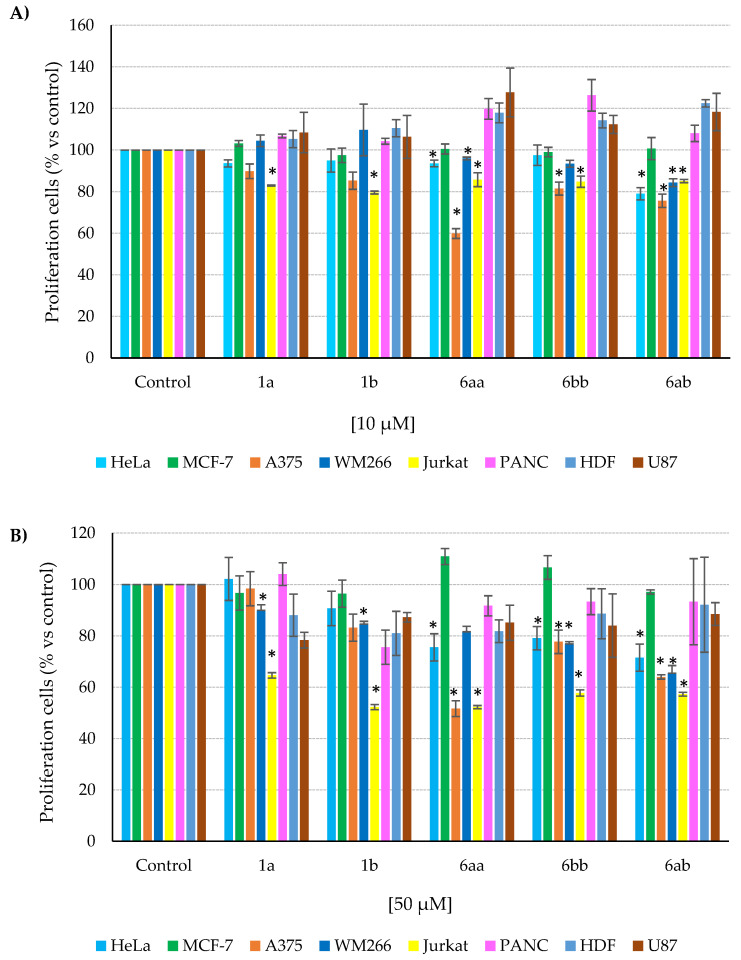
Effect of compounds on tumors and healthy cell proliferation. The cells were incubated in the presence of the compounds at 10 μM (**A**) or 50 μM (**B**) for 48 h at 37 °C. The results are presented as the percentage of proliferating cells with respect to the control (vehicle-treated cells) and are expressed as means ± SE, * *p* < 0.05.

In this context, the antiproliferative effect of silibinin compounds was evaluated on many human tumor cell lines of disparate histological origins or different metastatic potential, and human dermal fibroblasts (HDFs) were used as healthy cells to evaluate the selectivity of action of the examined compounds towards tumor cells. A preliminary screening was performed by treating cells with 10 and 50 μM concentrations for 48 h to identify the most sensitive cell lines. The results show that silibinin compounds were active on Jurkat, A375, WM266, and HeLa, but at the tested concentrations, they did not interfere with the growth of PANC, MCF-7, HDF or U87 (Figure 2). This reveals an interesting tumor cell selectivity, an important feature for the optimization of therapeutic compounds. All molecules showed good activity on Jurkat cells derived from leukemia, even at the lowest used concentration (10 μM), displaying a reduction in proliferation of about 20%, which reached more than 40% when the cells were incubated with the molecules at 50 μM. Interestingly, the dimers appeared to be more active than monomers on the melanoma cell lines used (WM266 and A375) (Figure 2).

**Figure 3:**
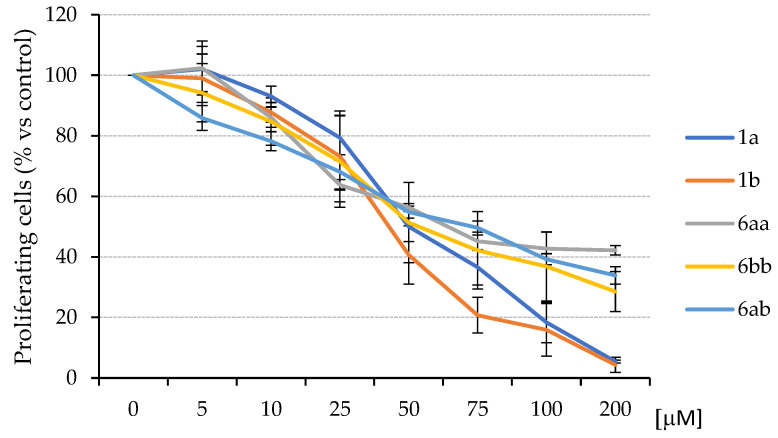
Dose–response curves obtained using the indicated concentrations of compounds on responsive cells. The proliferation was determined by CCK8 assay. The results are presented as the percentage of proliferating cells compared with the control (vehicle-treated cells) and are expressed as means ± SE of two independent experiments performed in triplicate.

For further studies, dose–response curves were obtained (Figure 3), and the corresponding IC_50_ values were calculated on the cells resulted more sensitive to the treatment with the compounds, the leukemia cell line Jurkat. As shown in Table 2, IC_50_ values were quite similar for all tested molecules. Nevertheless, the most active compound was found to be SilB, with an IC_50_ of 36 μM. Importantly, all molecules were poorly active on HDFs, showing their selectivity of action towards tumor cells. In particular, monomers showed an IC_50_ of about 200 μM, and dimers were even more selective with higher IC_50_ (Figure 3). These data are interesting considering that, in similar experiments, silibinin compounds are usually used in concentrations of up to 300 μM [27]. A relevant outcome is the identification of a molecule with low toxicity in healthy cells. As reported in the literature, silibinin exerts its cytotoxic effect by activating the apoptotic pathway [28,29]; therefore, we investigated whether dimers induce apoptosis to the same degree as the related monomers.

For this purpose, Jurkat was incubated with the molecules at a concentration of 200 μM, and flow cytometric analysis with annexin V/propidium iodine (PI) double staining was carried out.

**Figure 4:**
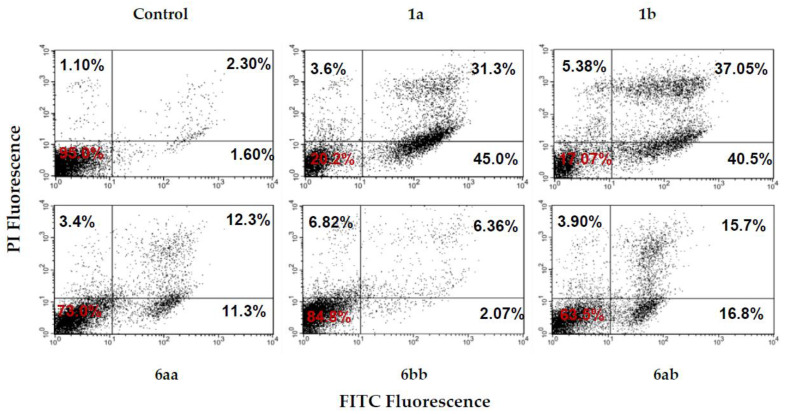
Apoptosis analysis with annexin V-FITC/PI double-staining method on Jurkat cells. The cells were treated with the indicated compounds (**1a**, **1b**, **6aa**, **6bb** and **6ab**) at a concentration of 200 μM at 37 °C for 48 h. The control (**Control**) is the vehicle-treated sample. In each panel, lower left quadrant: viable cells; upper left: necrotic cells; upper right: advanced apoptotic cells; lower right: early apoptotic cells. This picture is representative of two independent experiments.

The results indicate that **6aa** and **6ab** could induce apoptosis as effectively as the monomers, although they showed a lower percentage of apoptotic cells; in particular, 20% of cells treated with **6aa** were apoptotic (early and advanced) with respect to the control, and **6ab** exhibited about 25% (early and advanced) apoptotic cells, whereas 70% of cells treated with **1a** or **1b** were apoptotic (Figure 4). In contrast, **6bb** was not able to induce apoptosis: only 4% of treated cells were apoptotic compared with the control (Figure 4). These results indicate not only that the dimers probably have a different mechanism of action from the monomers but also that the behaviour differs among the dimers, underlining the importance of the stereochemistry of the molecules and suggesting that it could affect the activity of the silybin compounds by means of specific and selective interactions with protein partners.

## 3. Materials and Methods

### 3.1. General Methods and Materials

All chemicals were purchased from Sigma–Aldrich (Milano, Italy). HPLC–grade MeCN and MeOH were purchased from Carlo Erba Reagents and Sigma-Aldrich, respectively. Reactions were monitored by TLC (F254 precoated silica gel plates, Merck) and column chromatography (Merck Kieselgel 60, 70–230 mesh, Milano, Italy). HPLC analysis of dimers **6aa**, **6bb** and **6ab**, was performed with a Shimadzu LC–8A PLC system (Shimadzu Analytical and Measuring Instruments, Milano, Italy) equipped with a Shimadzu SCL–10A VP System control and a Shimadzu SPD–10A VP UV–Vis detector. Mass spectrometric analyses were performed on AB SCIEX TOF/TOF 5800 in positive or negative mode and Waters Micromass ZQ Instrument (Waters, Milano, Italy) equipped with an electrospray source in positive mode. The NMR spectra were recorded at 25 °C on an NMR spectrometer Bruker DRX, Bruker Advance (Bruker Italia Srl, Milano, Italy) and INOVA-500 NMR instrument (Varian, Milan, Italy), referenced in ppm to residual solvent signals (CDCl_3_, at δ_H_ 7.27, δ_C_ 77.0; CD_3_OD, at δ_H_ 3.31, δ_C_ 49.0 and DMSO-d_6_, δ_H_ 2.50, δ_C_ 39.5. ^31^P NMR spectra were recorded using D_3_PO_4_ (85 wt. % in D_2_O, 98 atoms %D) as an external standard, referenced to residual solvent signals (δ_P_ 0.0 ppm). Data for ^1^H NMR are reported as follows: chemical shift (ppm), multiplicity (s = singlet, br = broad, d = doublet, t = triplet, and m = multiplet), coupling constant (Hz), integration, and assignment. The ^1^H signals were assigned by using ^1^H/^1^H COSY, ^1^H/^13^C HSQC, and ^1^H/^13^C HMBC. NMR data were processed using Bruker Topspin 3.6.1 software. The proton-detected heteronuclear correlations were measured using a gradient heteronuclear single-quantum coherence (HSQC) experiment, optimized for ^1^J_HC_ = 155 Hz, and a gradient heteronuclear multiple bond coherence (HMBC) experiment, optimized for ^n^J_HC_ = 8 Hz.

Silybin A and silybin B were obtained by HPLC purification of silibinin purchased from Sigma–Aldrich (S0417) as reported by us [21]. The experimental procedures to the synthesis of building blocks **2** and **4**, are described in detail only for the stereoisomers of silybin A: the same reaction conditions (temperature, stoichiometric ratios, time of reaction) were used for silybin B.

### 3.2. Synthesis of 3,5,7,4′′-O-tetra-isobutyryl-silybin **2a** (or **2b**)

Silybin A (**1a**, 730 mg, 1.51 mmol), previously co-evaporated several times with anhydrous THF and dissolved in anhydrous pyridine (4 mL), was reacted with DMTCl (666 mg, 1.96 mmol). The reaction mixture, left at room temperature for 2 h under stirring, was then diluted with MeOH and concentrated under reduced pressure. The crude was then diluted with DCM, transferred into a separatory funnel, washed once with a saturated NaHCO_3_ aqueous solution, and then once with H_2_O. The organic phase, dried over anhydrous Na_2_SO_4_, was filtered, and then concentrated under reduced pressure. The crude was next purified on a silica gel column, eluting with DCM/MeOH (98:2, *v*/*v*) in the presence of 1% of pyridine, affording pure 9′′-*O*-(4,4′-dimethoxytriphenylmethyl)-silybin A as a pale amorphous solid (1.14 g, 1.44 mmol) in a 96% yield.

In total, 1.14 g (1.44 mmol) of product dissolved in anhydrous DCM (20 mL), adding Et_3_N (841 μL, 6.05 mmol) and pyridine (1.2 mL, 14.4 mmol), was reacted with isobutyryl chloride (638 μL, 6.05 mmol). The mixture was left under stirring at 0 °C for 15 min and then diluted with MeOH and concentrated under reduced pressure. The crude was then diluted with DCM, transferred into a separatory funnel, washed one time with a saturated NaHCO_3_ aqueous solution, and then once with H_2_O. The organic phase, dried over anhydrous Na_2_SO_4_, was filtered, and then concentrated under reduced pressure. The crude was next purified on a silica gel column, eluting with *n*-hexane/EtOAc (7:3, *v*/*v*) in the presence of 1% of pyridine furnishing pure 3,5,7,4′′-*O*-tetra-isobutyryl-9′′-*O*-(4,4′-dimethoxytriphenylmethyl)-silybin A as pale amorphous solid (1.06 g, 1.0 mmol) in a 70% yield.

In a solution, 0.1 M of product (1.06 g, 1.0 mmol) in MeOH/DCM (6:1 *v*/*v*) was added 1% (p/v) of I_2_ (100 mg). The solution was left under stirring at room temperature for 1 h and then added Na_2_S_2_O_3_ and concentrated under reduced pressure. The crude was then diluted with DCM, transferred into a separatory funnel, washed one time with a saturated NaHCO_3_ aqueous solution, and then once with H_2_O. The organic phase, dried over anhydrous Na_2_SO_4_, was filtered, and then concentrated under reduced pressure. The crude was next purified on a silica gel column, eluting with *n*-hexane/EtOAc (6:4, *v*/*v*), leading pure 3,5,7,4′′-*O*-tetra-isobutyryl-silybin A (**2a**) as pale amorphous solid (686 mg, 0.9 mmol) in a 90% yield.

**2a** 690 mg (60% starting from **1a**). Rf = 0.5 (*n*-hexane/EtOAc 6:4 *v*/*v* silica gel). ^1^H-NMR (400 MHz, CDCl_3_, 25 °C, δ ppm, *J* Hz): 7.13–6.96 (complex signals, 6H, H-2′, H-5′, H-6′, H-2′′, H-5′′, H-6′′); 6.74 (d, *J* = 2.2, 1H, H-6); 6.55 (d, *J* = 2.2, 1H, H-8); 5.66 (d, *J* = 12.2, 1H, H-3); 5.35 (d, *J* = 12.2, 1H, H-2); 5.02 (d, *J* = 8.0, 1H, H-7′′); 4.02–3.97 (m, 1H, H8′′); 3.87–3.82 (overlapped signals, 4H, OCH_3_ and H-9′′a); 3.57 (dd, *J* = 12.5, 3.6, 1H, H-9′′b); 2.97–2.71 (m, 3H, CH of isobutyryl (*i*bu) groups in 5, 7 and 4′′); 2.59–2.49 (m, 1H, CH of *i*bu in 3); 1.37–1.22 (m, 18H, CH_3_ of *i*bu groups in 5, 7 and 4′′); 1.12–0.97 (m, 6H, CH_3_ of *i*bu group in 3). ^13^C NMR (100 MHz, CDCl_3_, 25 °C, δ ppm): 185.0; 175.3; 175.0 (2C); 174.1; 162.4; 156.5; 151.7; 151.6; 144.1; 143.6; 140.5; 134.5; 128.6; 123.0; 120.8; 119.8; 117.0, 116.5, 111.1 (2C); 110.7; 108.7; 81.1; 78.3; 76.0; 72.9; 61.4; 56.1; 34.2; 34.0; 33.9; 33.6; 19.0; 18.8; 18.7; 18.5. MS (MALDI-TOF, positive ions): *m/z* calculated for C_41_H_46_O_14_ = 762.289; found: 763.996 [M + H]^+^, 785.229 [M + Na]^+^, 801.652 [M + K]^+^.

**2b** 644 mg (55% starting from **1b**). Rf = 0.5 (*n*-hexane/EtOAc 6:4 *v*/*v* silica gel). ^1^H NMR (400 MHz, CDCl_3_, 25 °C, δ ppm, *J* Hz): 7.11–6.96 (complex signals, 6H, H-2′, H-5′, H-6′, H-2′′, H-5′′, H-6′′); 6.75 (d, *J* = 2.2, 1H, H-6); 6.55 (d, *J* = 2.2, 1H, H-8); 5.67 (d, *J* = 12.1, 1H, H-3); 5.36 (d, *J* = 12.2, 1H, H-2); 5.04 (d, *J* = 8.0, 1H, H-7′′); 4.02–3.97 (m, 1H, H8′′); 3.86–3.80 (overlapped signals, 4H, OCH_3_ and H-9′′a); 3.56 (dd, *J* = 12.4, 3.6, 1H, H-9′′b); 2.96–2.73 (m, 3H, CH of *i*bu groups in 5, 7 and 4′′); 2.60–2.50 (m, 1H, CH of *i*bu group in 3); 1.35–1.26 (m, 18H, CH_3_ of *i*bu groups in 5, 7 and 4′′); 1.02–0.92 (m, 6H, CH_3_ of *i*bu group in 3). ^13^C NMR (100 MHz, CDCl_3_, 25 °C, δ ppm): 185.0; 175.1; 175.0 (2C); 174.1; 162.4; 156.5; 151.7; 151.6; 144.1; 143.6; 140.6; 134.5; 128.5; 123.1; 120.9; 119.7; 117.1, 116.4, 111.3; 111.1; 110.8; 108.7; 81.0; 78.3; 75.9; 72.8; 61.4; 56.0; 34.2; 34.0; 33.9; 33.6; 19.0; 18.8; 18.7; 18.5. MS (MALDI-TOF, positive ions): *m*/*z* calculated for C_41_H_46_O_14_ = 762.289; found: 763.425 [M + H]^+^, 785.784 [M + Na]^+^, 801.358 [M + K]^+^.

### 3.3. Synthesis of Phosphoramidite Silybin 4a (or 4b)

To 3,5,7,4′′-tetra-*O*-*i*Bu-silybin A (**2a**, 0.26 mmol; 200 mg) dissolved in anhydrous DCM (4.5 mL), DIEA (181 μL, 1.05 mmol), and 2-cyanoethyl-N,N-diisopropylamino-chlorophosphoramidite **3** (73 μL, 0.31 mmol) were mixed. After 20 min the solution was concentrated and silica gel chromatography of the residue (eluent *n*-hexane/EtOAc 6:4, *v*/*v*, with 3% *v*/*v* of Et_3_N), afforded desired compound **4a** in 86% yield.

**4a** 219 mg (0.22 mmol, 86%). Rf = 0.8 (*n*-hexane/EtOAc 6:4, *v*/*v* silica gel). ^1^H NMR (400 MHz, CDCl_3_, 25 °C, δ ppm, *J* Hz, mixture of diastereoisomers): 7.11–6.94 (complex signals, 6H, H-2′, H-5′, H-6′, H-2′′, H-5′′, H-6′′); 6.74 (d, *J* = 2.2, 1H, H-6); 6.55 (d, *J* = 2.2, 1H, H-8); 5.66 (d, *J* = 12.2, 1H, H-3); 5.65 (d, *J* = 12.2, 1H, H-3); 5.35 (d, *J* = 12.2, 1H, H-2); 5.34 (d, *J* = 12.2, 1H, H-2); 5.02 (d, *J* = 7.8, 1H, H-7′′); 4.14–4.06 (m, 1H, H-8′′); 3.97–3.49 (m, 9H, OCH_3_, 2H-9′′, OCH_2_CH_2_CN, N[CH(CH_3_)_2_]_2_; 2.97–2.48 (m, 6H, CH of *i*bu groups, OCH_2_CH_2_CN); 1.36–1.26 (m, 18H, CH_3_ of *i*bu groups in 5, 7 and 4′′); 1.20–1.07 (m, 15H, CH_3_ of *i*bu groups in 3, N[CH(CH_3_)_2_]_2_). ^13^C NMR (100 MHz, CDCl_3_, 25 °C, δ ppm, mixture of diastereoisomers): 185.0 (2C); 175.2 (2C); 175.0 (2C); 174.1; 162.5; 156.5; 151.7; 151.5 (2C); 144.2; 143.5; 143.4; 140.5(2C); 134.6; 128.3; 128.2; 123.0; 122.9; 120.9; 120.8; 119.9; 119.8; 117.6; 117.5; 117.1; 116.9; 116.5; 116.4; 111.4; 111.2; 111.1; 110.7, 108.7; 81.2; 81.1; 76.2; 75.8; 72.9 (2C); 62.5; 62.2; 62.0; 58.7; 58.6; 58.5; 58.3; 56.0 (2C); 43.3; 43.2; 34.2; 34.0; 33.9; 33.6; 24.6; 24.5; 20.4; 20.3 (3C); 19.0; 18.8; 18.7; 18.5. ^31^P NMR (CDCl_3_, 161.98 MHz, 25 °C, δ ppm): 150.3; 149.9. MS (ESI-TOF, positive ions): *m*/*z* calculated for C_50_H_63_N_2_O_15_P = 962.397; found: 964.229 [M + H]^+^, 986.033 [M + Na]^+^, 1002.553 [M + K]^+^.

**4b** 202 mg (0.21 mmol, 80%). Rf = 0.8 (*n*-hexane/EtOAc 6:4, *v*/*v*, silica gel). ^1^H NMR (400 MHz, CDCl_3_, 25 °C, δ ppm, *J* Hz, mixture of diastereoisomers): 7.11–6.94 (complex signals, 6H, H-2′, H-5′, H-6′, H-2′′, H-5′′, H-6′′); 6.75 (d, *J* = 2.2, 1H, H-6); 6.55 (d, *J* = 2.2, 1H, H-8); 5.67 (d, *J* = 12.1, 1H, H-3); 5.35 (d, *J* = 12.1, 1H, H-2); 5.34 (d, *J* = 12.1, 1H, H-2); 5.02 (d, *J* = 7.8, 1H, H-7′′); 4.14–4.05 (m, 1H, H-8′′); 3.97–3.50 (m, 9H, OCH_3_, 2H-9′′, OCH_2_CH_2_CN, N[CH(CH_3_)_2_]_2_; 2.96–2.49 (m, 6H, CH of *i*bu groups, OCH_2_CH_2_CN); 1.36–1.26 (m, 18H, CH_3_ of *i*bu groups in 5, 7 and 4′′); 1.20–1.07 (m, 15H, CH_3_ of *i*bu groups in 3, N[CH(CH_3_)_2_]_2_) ppm. ^13^C NMR (100 MHz, CDCl_3_, 25 °C, δ ppm, mixture of diastereoisomers): 185.0; 175.1 (2C); 175.0; 174.9; 174.1; 162.4; 156.5; 151.7; 144.3; 144.2; 143.5 (2C); 140.5 (2C); 134.7; 128.2; 128.1; 123.0; 122.9; 121.0; 119.9; 119.8; 117.6; 117.5; 117.1; 116.5; 116.3; 111.1; 110.8, 108.7; 81.1; 81.0; 76.1; 75.8; 72.8; 62.7; 62.5; 62.2; 62.1; 58.7; 58.5 (2C); 58.3; 56.0 (2C); 43.3; 43.2; 34.2; 34.0; 33.9; 33.6; 24.6 (2C); 24.5 (2C); 20.3 (3C); 19.0; 18.8; 18.7 (3C); 18.5. ^31^P NMR (CDCl_3_, 161.98 MHz, 25 °C, δ ppm): 150.2; 149.8. MS (ESI-TOF, positive ions): *m*/*z* calculated for C_50_H_63_N_2_O_15_P = 962.397; found: 964.215 [M + H]^+^, 986,212 [M + Na]^+^, 1002.322 [M+K]^+^.

### 3.4. General Procedure for the Synthesis of Phosphotriester dimers 5aa, 5bb and 5ab

In total, 219 mg (0.22 mmol) of phosphoramidites **4a** and the building block **2a** 155 mg (0.20 mmol) previously dried and kept under reduced pressure, were reacted with a 0.25 M 4,5-dicyanoimidazole solution in anhydrous MeCN (1.5 mL, 0.37 mmol). To obtain the **5bb** dimer, the phosphoramidite **4b** and the derivative **2b** were coupled under the same conditions as previously reported. To obtain the **5ab** dimer, the best yields were obtained by coupling the phosphoramidite **4a** and the derivative **2b**. The reaction was left under stirring at r.t. and monitored by TLC with an eluent system *n*-hexane/EtOAc (6:4, *v*/*v*). After 30 min, the reaction was over, and then a 5.5 M *tert*-Butyl hydroperoxide (TBHP) solution in decane (150 μL) was added and left stirring at r.t. After 30 min the reaction mixture was, concentrated under reduced pressure, and purified by flash chromatography, eluting with *n*-hexane/EtOAc (7:3, *v*/*v*), to afford pure **5** (**5aa**, **5bb** and **5ab**) yellow-brown amorphous powder in 83%, 80% and 77% yields, respectively.

**5aa** 272 mg (0.17 mmol, 83%). Rf = 0.5 (*n*-hexane/EtOAc 1:1, *v*/*v*, silica gel). ^1^H-NMR (400 MHz, CDCl_3_, 25 °C, δ ppm, *J* Hz): 7.11–6.91 (complex signals, 12H, H-2′, H-5′, H-6′, H-2′′, H-5′′, H-6′′); 6.76 (s, 2H, H-6); 6.55 (s, 2H, H-8); 5.66 (d, *J* = 12.1, 2H, H-3); 5.36 (d, *J* = 12.1, 2H, H-2); 4.93 (t, *J* = 8.0, 2H, H-7′′); 4.32–3.95 (m, 8H, H8′′, 2H9′′, OCH_2_CH_2_CN); 3.86–3.80 (overlapped signals, 6H, OCH_3_); 2.95–2.49 (m, 10H, CH of *i*bu groups, OCH_2_CH_2_CN); 1.39–1.25 (m, 18H, CH_3_ of *i*bu groups in 5, 7 and 4′′); 1.14–1.08 (m, 6H, CH_3_ of *i*bu groups in 3); 1.01–0.96 (m, 6H, CH_3_ of *i*bu groups in 3). ^13^C NMR (100 MHz, CDCl_3_, 25 °C, δ ppm): 184.9; 175.1; 175.0; 174.1; 162.4; 156.5; 151.7; 143.6; 143.4; 143.3; 140.8; 133.7; 128.9; 123.3; 123.2; 121.3; 121.1; 119.8 (2C); 117.2; 117.1; 116.6; 116.5; 116.4; 111.5, 111.4; 111.1; 110.7, 108.7; 80.9; 76.0; 75.9; 75.7; 72.8; 72.7; 66.5; 62.3; 56.0; 34.2; 34.0; 33.9; 33.6; 19.0; 18.8; 18.7 (C2); 18.5. ^31^P NMR (161 MHz, CDCl_3_, 25 °C, δ ppm): -2.4. MS (MALDI-TOF, positive ions): *m*/*z* calculated for C_85_H_94_NO_30_P = 1639.560; found: 1641.838 [M+H]^+^, 1663.652 [M + Na]^+^, 1679.154 [M + K]^+^.

**5bb** 262 mg (0.16 mmol, 80%). Rf = 0.5 (*n*-hexane/EtOAc 1:1, *v*/*v*, silica gel). ^1^H NMR (400 MHz, CDCl_3_, 25 °C, δ ppm, *J* Hz): 7.11–6.91 (complex signals, 12H, H-2′, H-5′, H-6′, H-2′′, H-5′′, H-6′′); 6.76 (s, 2H, H-6); 6.55 (s, 2H, H-8); 5.66 (d, *J* = 12.1, 2H, H-3); 5.36 (d, *J* = 12.1, 2H, H-2); 4.93 (t, *J* = 8.0, 2H, H-7′′); 4.32–3.95 (m, 8H, H8′′, 2H9′′, OCH_2_CH_2_CN); 3.86–3.80 (overlapped signals, 6H, OCH_3_); 2.95–2.49 (m, 10H, CH of *i*bu groups, OCH_2_CH_2_CN); 1.36–1.24 (m, 18H, CH_3_ of *i*bu groups in 5, 7 and 4′′); 1.13–1.09 (m, 6H, CH_3_ of *i*bu groups in 3); 1.02–0.98 (m, 6H, CH_3_ of *i*bu groups in 3) ppm. ^13^C-NMR (125 MHz, CDCl_3_, 25 °C, δ ppm, *J* Hz): 184.9; 175.1; 175.0 (2C); 174.1; 162.4; 156.5; 151.7 (2C); 143.6; 143.4; 143.3; 140.8; 133.7; 128.9; 123.3 (2C); 121.2; 121.1; 119.8 (2C); 117.2; 117.1; 116.6 (2C); 116.4; 111.5, 111.4; 111.1; 110.7, 108.7; 80.9; 75.9; 75.7; 72.8; 66.5 (2C); 62.3; 62.2; 56.0; 34.2; 34.0; 33.9; 33.6; 19.0; 18.8; 18.7; 18.5. ^31^P NMR (161 MHz, CDCl_3_, 25 °C, δ ppm): -2.4. MS (MALDI-TOF, positive ions): *m*/*z* calculated for C_85_H_94_NO_30_P = 1639.560; found: 1641.554 [M + H]^+^, 1663,159 [M + Na]^+^, 1679.555 [M + K]^+^.

**5ab** 262 mg (0.15 mmol, 77%). Rf = 0.5 (*n*-hexane/EtOAc 1:1, *v*/*v*, silica gel). ^1^H NMR (400 MHz, CDCl_3_, 25 °C, δ ppm, *J* Hz): 7.12–6.88 (complex signals, 12H, H-2′, H-5′, H-6′, H-2′′, H-5′′, H-6′′); 6.76–6.73 (m, 2H, H-6); 6.56–6.54 (m, 2H, H-8); 5.69–5.61 (m, 2H, H-3); 5.38–5.32 (m, 2H, H-2); 4.99–5.90 (m, 2H, H-7′′); 4.35–3.97 (m, 8H, H8′′, 2H9′′, OCH_2_CH_2_CN); 3.88–3.79 (overlapped signals, 6H, OCH_3_); 2.96–2.48 (m, 10H, CH of *i*bu groups, OCH_2_CH_2_CN); 1.36–1.24 (m, 18H, CH_3_ of *i*bu groups in 5, 7 and 4′′); 1.13–1.08 (m, 6H, CH_3_ of *i*bu groups in 3); 1.02–0.96 (m, 6H, CH_3_ of *i*bu groups in 3) ppm. ^13^C NMR (100 MHz, CDCl_3_, 25 °C, δ ppm): 184.9; 175.1; 175.0 (2C); 174.1; 162.4; 156.5; 151.7 (2C); 143.6; 143.4; 143.3; 140.8; 133.7; 128.9; 123.3 (2C); 121.2; 121.1; 119.8 (2C); 117.2; 117.1; 116.6 (2C); 116.4; 111.5, 111.4; 111.1; 110.7, 108.7; 80.9; 76.1; 76.0; 75.9; 75.7; 72.8; 66.5 (2C); 62.3; 62.2; 56.0; 34.2; 34.0; 33.9; 33.6; 19.0; 18.8; 18.7 (2C); 18.5. ^31^P NMR (161 MHz, CDCl_3_, 25 °C, δ ppm): -2.3; -2.6. MS (MALDI-TOF, positive ions): *m*/*z* calculated for C_85_H_94_NO_30_P = 1639.560; found: 1641.497 [M + H]^+^, 1663.132 [M + Na]^+^, 1679.115 [M + K]^+^.

### 3.5. General Procedure for the Synthesis of Dimers 6aa, 6bb and 6ab

In total, 200 mg (0.12 mmol) of dimer **5aa** were treated with 7mL of a mixture conc. aq NH_3_/MeOH (1:1, *v*/*v*) for 5 h at 50 °C, leading to full removal of the *i*bu and 2-cyanoethyl (CE) groups. The mixture was dried under reduced pressure and suspended in a buffer solution and then purified on RP-HPLC carried out on Phenomenex Kromasil^®^ C18 column (10 μm particle size, 10.0 mm × 250 mm i.d.) using a linear gradient of MeCN in in 0.1 M Ammonium Acetate in H_2_O (pH 7.0) (pH 7.0) from 5% to 95% over 20 min at a flow rate of 6 mL/min with detection at 288, 260 nm. Compound thus obtained was converted into the corresponding sodium salts by cation exchange on a DOWEX (Na^+^ form) resin to obtain homogeneous samples **6aa** in 77% yield. RP-HPLC analysis was carried out on Luna C18 (2) (5 μm particle size, 150 mm × 4.6 mm i.d.) using a linear gradient of MeCN in in 0.1 M ammonium acetate in H_2_O (pH 7.0) from 5% to 95% over 20 min at a flow rate of 0.8 mL/min with detection at 288 nm. The purity of the **6aa** product was 99.6% (see SM).

**6aa** 97 mg (0.09 mmol, 77%). t_R_ = 13.3 min (99.6% purity, see Figure S1 in SM). ^1^H NMR (400 MHz, DMSO-d6 + 5% D_2_O, 25 °C, δ ppm, *J* Hz); 7.11–6.74 (complex signals, 12H, H-2′, H-5′, H-6′, H-2′′, H-5′′, H-6′′) 5.85 (s, 4H, H-6 and H-8); 5.05 (d, *J* = 11.0, 2H, H-2); 4.82 (d, *J* = 7.6, 2H, H-7′′); 4.57 (d, *J* = 11.0, 1H, H-3); 4.35–4.27 (m, 2H, H8′′); 3.80–3.65 (overlapped signals, 8H, OCH_3_, H-9′′a); 3.58– 3.47 (m, 2H, H-9′′b) ppm. ^13^C NMR (100 MHz, DMSO-d6 + 5% D_2_O, 25 °C, δ ppm): 197.8; 168.2; 163.7; 162.8; 148.0; 147.5; 143.8; 143.6; 130.5; 127.6; 121.8; 120.8; 116.8; 116.7; 115.7; 112.1, 100.6; 96.7; 95.7; 82.9; 77.1; 76.2; 71.8; 63.6; 56.0. ^31^P NMR (161 MHz, DMSO-d6 + 5% D_2_O, 25 °C, δ ppm): -1.6. MS (MALDI-TOF, negative ions): *m*/*z* calculated for C_50_H_43_O_22_P = 1026.198; found: 1025.355 [M − H]^-^.

**6bb** 103 mg (0.10 mmol, 82%). t_R_ = 13.4 min (98.7% purity, see Figure S2 in SM). ^1^H NMR (400 MHz, DMSO-d6, 25 °C, δ ppm, *J* Hz): 11.90 (s, 2H, OH-5); 11.08 (s, 2H, OH-7); 9.19 (s, 2H, OH-4′′); 7.10–6.73 (complex signals, 12H, H-2′, H-5′, H-6′, H-2′′, H-5′′, H-6′′) 5.91 (s, 4H, H-6 and H-8); 5.85–5.79 (m, 2H, OH-3); 5.05 (d, *J* = 11.1, 2H, H-2); 4.83 (d, *J* = 7.8, 2H, H-7′′); 4.60 (d, *J* = 11.1, 1H, H-3); 4.58 (d, *J* = 11.1, 1H, H-3); 4.34–4.26 (m, 2H, H8′′); 3.79–3.65 (overlapped signals, 8H, OCH_3_, H-9′′a); 3.58– 3.48 (m, 2H, H-9′′b) ppm. ^13^C NMR (100 MHz, DMSO-d6, 25 °C, δ ppm, *J* Hz): 198.2; 167.4; 163.7; 162.9; 148.0; 147.5; 143.8; 143.5; 130.5; 127.6; 121.6; 120.8; 117.0; 116.8; 115.7; 112.1, 100.8; 96.5; 95.5; 83.0; 77.1; 76.2; 71.9; 63.6; 56.0. ^31^P NMR (161 MHz, DMSO-d6, 25 °C, δ ppm): -1.7. MS (MALDI-TOF, negative ions): *m*/*z* calculated for C_50_H_43_O_22_P = 1026.198; found: 1025.196 [M − H]^−^.

**6ab** 100 mg (0.10 mmol, 80%). t_R_ = 13.5 min (99.2% purity, see Figure S3 in SM). ^1^H NMR (400 MHz, MeOD-d4, 25 °C, δ ppm, *J* Hz): 7.15–6.79 (complex signals, 12H, H-2′, H-5′, H-6′, H-2′′, H-5′′, H-6′′) 5.92 (s, 4H, H-6 and H-8); 5.02–4.94 (m, 4H, H-2 and H7′′); 4.53 (d, 11.5, 1H, H-3); 4.26–4.20 (m, 2H, H8′′); 3.91–3.80 (overlapped signals, 8H, OCH_3_, H-9′′a); 3.79– 3.75 (m, 2H, H-9′′b) ppm. ^13^C NMR (100 MHz, MeOD-d4, 25 °C, δ ppm): 196.9; 167.3; 163.9; 162.9; 147.7; 146.9; 143.9; 143.8; 143.6; 130.0; 127.8; 120.6; 120.3; 116.5; 116.3; 116.1; 114.9; 110.9, 100.4; 96.9; 94.9; 83.3 (2C); 77.0; 76.1; 76.0; 72.2; 64.0; 55.1. ^31^P NMR (161 MHz, MeOD-*d*_4_, 25 °C, δ ppm, *J* Hz): -0.2. MS (MALDI-TOF, negative ions): *m*/*z* calculated for C_50_H_43_O_22_P = 1026.198; found: 1025.144 [M − H]^−^.

### 3.6. Hydroxyl Radical (∙OH) Generation and Reactivity Estimation

The reactivity constant between new silybin dimers and hydroxyl radical has been determined using Laser flash Photolysis system. The spectroscopic equipment and method have been described elsewhere, and only an introduction is given below [8].

The formation of di-thiocyanate radical anion (SCN_2_^●−^) through reactivity of photogenerated hydroxyl radicals (HO^●^) with thiocyanate (SCN^-^ in the presence of hydrogen peroxide. The second-order rate constants between HO^●^ and dimers were determined following reactions (R1–R4 in the Section 2.2) and using the following equation:

(1) $$\frac{{\mathrm{Abs}}_{0}}{\mathrm{Abs}}=1+\frac{k_{{\mathrm{HO}}^{•},\mathrm{Dimer}}^{\mathrm{II}}\left[\mathrm{Dimer}\right]}{k_{{\mathrm{HO}}^{•}{,\mathrm{SCN}}^{-}}^{\mathrm{II}}{[\mathrm{SCN}}^{-}]}$$

where Abs_0_ and Abs are the absorption of ${\mathrm{SCN}}_{2}^{•-}$ at 475 nm in absence and presence of dimers; $k_{{\mathrm{HO}}^{•}{,\mathrm{SCN}}^{-}}^{\mathrm{II}}$ and $k_{{\mathrm{HO}}^{•},\mathrm{Dimer}}^{\mathrm{II}}$ are the second-order rate constants of HO^●^ with thiocyanate and dimers at different concentrations. The plot of $\frac{{\mathrm{Abs}}_{0}}{\mathrm{Abs}}$ vs. the concentration of dimers (**6aa**, **6bb** or **6ab**) can be fitted with a liner correlation and slope used to determine the value of $k_{{\mathrm{HO}}^{•},\mathrm{Dimer}}^{\mathrm{II}}$. The results presented here were the mean of three replicates. Means were compared by one way analysis of variance (ANOVA) and significant differences were assessed by post hoc tests of least significant. Differences with a *p* value of < 0.05 were considered significance.

### 3.7. Culture Conditions

Human T lymphoblastoid (Jurkat) and human metastatic melanoma (WM266) cell lines were grown in RPMI medium supplemented with heat inactivated 10% fetal bovine serum (FBS), 2.5 mM glutamine, 100 U/mL penicillin, and 100 μg/mL streptomycin (Euroclone). Human cervix adenocarcinoma cell line (HeLa), human lung carcinoma (A549), human pancreatic cancer (PANC), human glioblastoma (U87) normal human fibroblasts (HDF) were grown in DMEM supplemented with 10% fetal bovine serum (FBS), 1% glutamine, 100 U/mL penicillin and 100 μg/mL streptomycin (Euroclone, Milano, Italy). Cells were maintained in humidified air containing 5% CO_2_, at 37 °C [30].

### 3.8. Antiproliferative Activity

Cells were plated at density of 10,000 cells/well for Jurkat, 2000/well for WM266 and HDF, 1200 cells/well for HeLa, and 1000 for PANC, U87 and A375 in 96-well microplates (Thermofisher, Waltham, MA, USA). After 24 h incubation, cells were treated with increasing concentrations of synthetized compounds previously solubilized in DMSO at 50 mM concentration. Cell proliferation was determined by using (2-(2-methoxy-4-nitrophenyl)-3-(4-nitrophenyl)-5-(2,4-disulfophenyl)-2H-tetrazolium, monosodium salt (CCK-8, Sigma Aldrich) for Jurkat cells [31], and the 3-(4,5-dimethylthiazol-2-yl)-2,5- diphenyltetrazolium bromide assay (MTT, Sigma Aldrich, Sigma Aldrich, St. Louis, MO, USA)) for HeLa, WM266, PANC, U87 and HDF cells [32], after 48 h treatment. Plates were then analyzed by using a microplate reader (Enspire, Perkin Elmer Italia Spa, Milano, Italy) at 450 (CCK-8) or 570 nm (MTT).

The results are presented as the percentage of proliferating cells respect to the control (vehicle treated cells) and are expressed as means ± SE of, at least, three independent experiments performed in triplicate. The statistical analysis was performed using Student’s *t*-test, unpaired, two-sided, *p* < 0.05 was considered significant. The IC_50_ values were calculated by GraphPad Prism software.

### 3.9. Apoptosis Assay

The apoptosis analysis was performed on Jurkat cells seeded at 2.5 × 10^5^ cells/mL in a 6-well plate. The cells were incubated in the absence or presence of 200 μM concentration of examined compounds at 37 °C and apoptosis induction was analysed after 48 h by double staining with annexin V/FITC and propidium iodide (PI) (eBioscience, Affimetrix Santa Clara, CA, USA) [33]. The cells undergoing apoptosis were quantified using a flow cytometer equipped with a 488 nm argon laser (Becton Dickinson, Franklin Lakes, NJ, USA) by Cell Quest software. All FACS analyses were performed at least 2 times.

## 4. Conclusions

In this work, we reported the synthesis of the optically pure phosphodiester dimers of silybins following a very efficient synthetic strategy using an orthogonal protection of the different OH groups. Starting from the two pure diastereoisomers and exploiting the well-known phosphoramidite chemistry, the new 9′′-9′′ dimers of silybin A and silybin B (**6aa**, **6ab** and **6bb**) were obtained in pure form and in good yields.

Their ability to scavenge reactive oxygen species (ROS) such as hydroxyl radical (HO^●^) highlights the high activity of all three dimers, comparable to that reported for a known potent antioxidant as quercetin. Although they are diastereomers, **6aa**, **6ab** and **6bb** show very similar radical scavenger activity.

To disclosing a structure–activity relationship, dimers (**6aa**, **6ab** and **6bb**), as well as the silybin A (**1a**) and silybin B (**1b**), a preliminary screening was performed by treating cells with 10 and 50 μM concentrations for 48 h. The results indicate that both monomers and dimers present selective anti-proliferative activity towards leukemia cells at the concentrations used, in particular, all silybin compounds showing a similar IC_50_ in the mid-micromolar range and are poorly active on normal cells. However, the mechanism by which the different silybin compounds induce their cytotoxic activity appears to be different: all the cells treated with the monomers go completely into apoptosis, whereas only part of the cells treated with **6aa** and **6ab** were found to be in apoptosis. Contrarily, when the cells were treated with **6bb** dimer, no significant number of cells in the apoptotic stage was observed. These results demonstrate the crucial role of the stereochemistry for these flavonolignans, in the activation of the apoptotic mechanism and opens up a new window to deeply investigate the interaction of such compounds with proteins involved in cancer metabolic pathways.

## Figures and Tables

**Table 1:** Second order rate constant $k_{{\mathrm{HO}}^{•},X}^{\mathrm{II}}$ of silybin phosphodiester dimers **6aa**, **6bb** and **6ab**.

Compound	$k_{HO^{•},X}^{II}\;(M^{-1}\cdot s^{-1}{)\;}^{a}$
**6aa**	8.63 ± 1.35 × 10^9^
**6bb**	1.01 ± 0.16 × 10^10^
**6ab**	1.48 ± 0.09 × 10^10^

^a^: calculated in H_2_O.

**Table 2:** IC_50_ values of compounds after 48 h incubation.

IC_50_ ± DS (μM)					
	SilA (1a)	SilB (1b)	SilAA (6aa)	SilBB (6bb)	SilAB (6ab)
**Jurkat**	46.7 ± 14.1	36 ± 14.3	71 ± 15.5	59 ± 19.9	64 ± 10.8
**HDF**	199 ± 34.5	172 ± 39.0	>200	>200	>200

## Data Availability

Data are contained within the article or Supplementary Materials (SM).

## Supplementary Materials

The following supporting information can be downloaded: HPLC profile of dimers **6aa**, **6bb** and **6ab** in Figures S1–S3; ^1^H, ^13^C and ^31^P NMR spectra of compounds **2**, **4****–6** in Figures S3–S31.

## Sample Availability

Samples of the compounds are not available from the authors.

## References

[1] Neha K., Haider M.R., Pathak A., Yar M.S. (2019). Medicinal prospects of antioxidants: A review. Eur. J. Med. Chem..

[2] Rosini M., Simoni E., Milelli A., Minarini A., Melchiorre C. (2014). Oxidative Stress in Alzheimer’s Disease: Are We Connecting the Dots?. J. Med. Chem..

[3] Quideau S., Deffieux D., Douat-Casassus C., Pouységu L. (2011). Plant Polyphenols: Chemical Properties, Biological Activities, and Synthesis. Angew. Chemie Int. Ed..

[4] Berube G. (2006). Natural and Synthetic Biologically Active Dimeric Molecules: Anticancer Agents, Anti-HIV Agents, Steroid Derivatives and Opioid Antagonists. Curr. Med. Chem..

[5] Thapa A., Woo E.-R., Chi E.Y., Sharoar M.G., Jin H.-G., Shin S.Y., Park I.-S. (2011). Biflavonoids Are Superior to Monoflavonoids in Inhibiting Amyloid-β Toxicity and Fibrillogenesis via Accumulation of Nontoxic Oligomer-like Structures. Biochemistry.

[6] Wong I.L.K., Zhu X., Chan K.F., Law M.C., Lo A.M.Y., Hu X., Chow L.M.C., Chan T.H. (2018). Discovery of Novel Flavonoid Dimers to Reverse Multidrug Resistance Protein 1 (MRP1, ABCC1) Mediated Drug Resistance in Cancers Using a High Throughput Platform with “Click Chemistry”. J. Med. Chem..

[7] Romanucci V., Di Fabio G., Zarrelli A. (2018). A New Class of Synthetic Flavonolignan-Like Dimers: Still Few Molecules, but with Attractive Properties. Molecules.

[8] Romanucci V., Gravante R., Cimafonte M., Di Marino C., Mailhot G., Brigante M., Zarrelli A., Fabio G. (2017). Di Phosphate-Linked Silibinin Dimers (PLSd): New Promising Modified Metabolites. Molecules.

[9] Lee D.Y.-W., Liu Y. (2003). Molecular Structure and Stereochemistry of Silybin A, Silybin B, Isosilybin A, and Isosilybin B, Isolated from *Silybum marianum* (Milk Thistle). J. Nat. Prod..

[10] Biedermann D., Vavříková E., Cvak L., Křen V. (2014). Chemistry of silybin. Nat. Prod. Rep..

[11] Gazak R., Walterova D., Kren V. (2007). Silybin and Silymarin—New and Emerging Applications in Medicine. Curr. Med. Chem..

[12] Polachi N., Bai G., Li T., Chu Y., Wang X., Li S., Gu N., Wu J., Li W., Zhang Y. (2016). Modulatory effects of silibinin in various cell signaling pathways against liver disorders and cancer—A comprehensive review. Eur. J. Med. Chem..

[13] Křen V. (2021). Chirality Matters: Biological Activity of Optically Pure Silybin and Its Congeners. Int. J. Mol. Sci..

[14] Romanucci V., Agarwal C., Agarwal R., Pannecouque C., Iuliano M., De Tommaso G., Caruso T., Di Fabio G., Zarrelli A. (2018). Silibinin phosphodiester glyco-conjugates: Synthesis, redox behaviour and biological investigations. Bioorg. Chem..

[15] Zarrelli A., Romanucci V., Tuccillo C., Federico A., Loguercio C., Gravante R., Di Fabio G. (2014). New silibinin glyco-conjugates: Synthesis and evaluation of antioxidant properties. Bioorg. Med. Chem. Lett..

[16] Di Fabio G., Romanucci V., De Nisco M., Pedatella S., Di Marino C., Zarrelli A. (2013). Microwave-assisted oxidation of silibinin: A simple and preparative method for the synthesis of improved radical scavengers. Tetrahedron Lett..

[17] Chambers C.S., Biedermann D., Valentová K., Petrásková L., Viktorová J., Kuzma M., Křen V. (2019). Preparation of Retinoyl-Flavonolignan Hybrids and Their Antioxidant Properties. Antioxidants.

[18] Zarrelli A., Romanucci V., De Napoli L., Previtera L., Di Fabio G. (2015). Synthesis of New Silybin Derivatives and Evaluation of Their Antioxidant Properties. Helv. Chim. Acta.

[19] Zarrelli A., Sgambato A., Petito V., De Napoli L., Previtera L., Di Fabio G. (2011). New C-23 modified of silybin and 2,3-dehydrosilybin: Synthesis and preliminary evaluation of antioxidant properties. Bioorg. Med. Chem. Lett..

[20] Zarrelli A., Romanucci V., Greca M., De Napoli L., Previtera L., Di Fabio G. (2012). New Silybin Scaffold for Chemical Diversification: Synthesis of Novel 23-Phosphodiester Silybin Conjugates. Synlett.

[21] Di Fabio G., Romanucci V., Di Marino C., De Napoli L., Zarrelli A. (2013). A Rapid and Simple Chromatographic Separation of Diastereomers of Silibinin and Their Oxidation to Produce 2,3-Dehydrosilybin Enantiomers in an Optically Pure Form. Planta Med..

[22] García-Viñuales S., Ahmed R., Sciacca M.F.M., Lanza V., Giuffrida M.L., Zimbone S., Romanucci V., Zarrelli A., Bongiorno C., Spinella N. (2020). Trehalose Conjugates of silybin as prodrugs for targeting toxic Aβ aggregates. ACS Chem. Neurosci..

[23] Sciacca M.F.M., Romanucci V., Zarrelli A., Monaco I., Lolicato F., Spinella N., Galati C., Grasso G., D’Urso L., Romeo M. (2017). Inhibition of Aβ Amyloid Growth and Toxicity by Silybins: The Crucial Role of Stereochemistry. ACS Chem. Neurosci..

[24] Rafat Husain S., Cillard J., Cillard P. (1987). Hydroxyl radical scavenging activity of flavonoids. Phytochemistry.

[25] Trouillas P., Marsal P., Svobodová A., Vostálová J., Gažák R., Hrbáč J., Sedmera P., Křen V., Lazzaroni R., Duroux J. (2008). Mechanism of the Antioxidant Action of Silybin and 2,3-Dehydrosilybin Flavonolignans: A Joint Experimental and Theoretical Study. J. Phys. Chem. A.

[26] Gažák R., Sedmera P., Vrbacký M., Vostálová J., Drahota Z., Marhol P., Walterová D., Křen V. (2009). Molecular mechanisms of silybin and 2,3-dehydrosilybin antiradical activity—Role of individual hydroxyl groups. Free Radic. Biol. Med..

[27] Guo S., Bai X., Liu Y., Shi S., Wang X., Zhan Y., Kang X., Chen Y., An H. (2021). Inhibition of TMEM16A by Natural Product Silibinin: Potential Lead Compounds for Treatment of Lung Adenocarcinoma. Front. Pharmacol..

[28] Ge Y., Zhang Y., Chen Y., Li Q., Chen J., Dong Y., Shi W. (2011). Silibinin Causes Apoptosis and Cell Cycle Arrest in Some Human Pancreatic Cancer Cells. Int. J. Mol. Sci..

[29] Si L., Liu W., Hayashi T., Ji Y., Fu J., Nie Y., Mizuno K., Hattori S., Onodera S., Ikejima T. (2019). Silibinin-induced apoptosis of breast cancer cells involves mitochondrial impairment. Arch. Biochem. Biophys..

[30] Capasso D., de Paola I., Liguoro A., Del Gatto A., Di Gaetano S., Guarnieri D., Saviano M., Zaccaro L. (2014). RGDechi-hCit: αvβ3 Selective Pro-Apoptotic Peptide as Potential Carrier for Drug Delivery into Melanoma Metastatic Cells. PLoS ONE.

[31] Iadonisi A., Traboni S., Capasso D., Bedini E., Cuomo S., Di Gaetano S., Vessella G. (2021). Switchable synthesis of glycosyl selenides or diselenides with direct use of selenium as the selenating agent. Org. Chem. Front..

[32] Pelliccia S., Amato J., Capasso D., Di Gaetano S., Massarotti A., Piccolo M., Irace C., Tron G.C., Pagano B., Randazzo A. (2020). Bio-Inspired Dual-Selective BCL-2/c-MYC G-Quadruplex Binders: Design, Synthesis, and Anticancer Activity of Drug-like Imidazo[2,1- i]purine Derivatives. J. Med. Chem..

[33] Capasso D., Di Gaetano S., Celentano V., Diana D., Festa L., Di Stasi R., De Rosa L., Fattorusso R., D’Andrea L.D. (2017). Unveiling a VEGF-mimetic peptide sequence in the IQGAP1 protein. Mol. Biosyst..

