# Supplementary material for: Phosphodiester Silybin Dimers Powerful Radical Scavengers: A Antiproliferative Activity on Different Cancer Cell Lines
Source: Molecules. 2022 Mar 5;27(5):1702. doi: 10.3390/molecules27051702 (PMC8911775; doi:10.3390/molecules27051702)

Phosphodiester silybin dimers powerful radical scavengers: A antiproliferative activity on different cancer cell lines

*Valeria Romanucci[1], Rita Pagano[1], Antonio Lembo[1], Domenica Capasso[2,3], Sonia Di Gaetano[2,4], Armando Zarrelli[1] and Giovanni Di Fabio[1,5]**

[1] Department of Chemical Sciences, University of Naples Federico II, Via Cintia 4, Napoli, 80126, Italy.

[2] CIRPEB, University of Naples "Federico II", Via Mezzocannone 16, Napoli, 80134, Italy.

[3] CESTEV, University of Naples "Federico II", Via De Amicis 95, Napoli, 80145, Italy.

[4] Institute of Biostructures and Bioimaging-CNR, Via Mezzocannone 16, Napoli, 80134, Italy.

[5] AIPRAS Onlus (Associazione Italiana per la Promozione delle Ricerche sull'Ambiente e la Salute umana Onlus) Via Campellone 50, Dugenta (BN), 82030, Italy.

*corresponding author email: difabio@unina.it

ELECTRONIC SUPPLEMENTARY INFORMATION:

Table of contents

Figure S1 RP-HPLC profile of dimer **6aa** ....pag 2
Figure S2 RP-HPLC profile of dimer **6aa** ...." 3
Figure S3 RP-HPLC profile of dimer **6aa** ...." 4
Figure S4 – S5: spectra of **2a** ...." 5-6
Figure S6 – S7: spectra of **2b** ...." 7-8
Figure S8 – S10: spectra of **4a** ...." 9-11
Figure S11 – S13: spectra of **4b** ...." 12-14
Figure S14 – S16: spectra of **5aa** ...." 15-17
Figure S17 – S19: spectra of **5bb** ...." 18-20
Figure S20 – S22: spectra of **5ab** ...." 21-23
Figure S23 – S25: spectra of **6aa** ...." 24-26
Figure S26 – S28: spectra of **6bb** ...." 27-29
Figure S29 – S31: spectra of **6ab** ...." 30-32

Figure S1 RP-HPLC analysis of dimer **6aa**

Channel A Results

Name	Time	Area	Area %	Conc (mg/L)
	13,02	16352	0,41	0,000
	13,37	3938752	99,59	0,000
Totals :		3955104	100,00	0,000

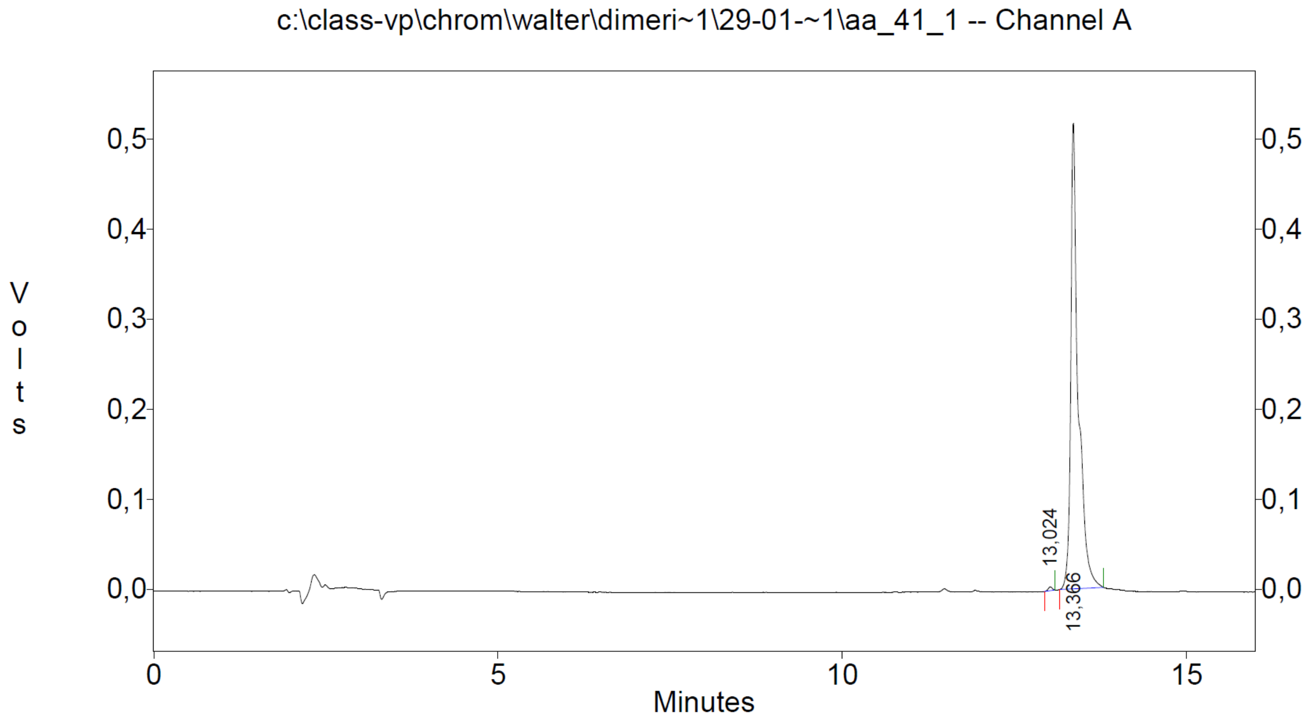

Linear gradient of MeCN in in 0.1 M ammonium acetate in H_2O (pH 7.0) from 5% to 95% over 20 min at a flow rate of 0.8 mL/min with detection at 288 nm.

Figure S2 RP-HPLC analysis of dimer **6bb**.

Channel A Results

Name	Time	Area	Area %	Conc (mg/L)
	12,01	17289	0,58	0,000
	13,14	12082	0,41	0,000
	13,41	2937685	98,66	0,000
	14,04	10401	0,35	0,000

Totals :

		2977457	100,00	0,000

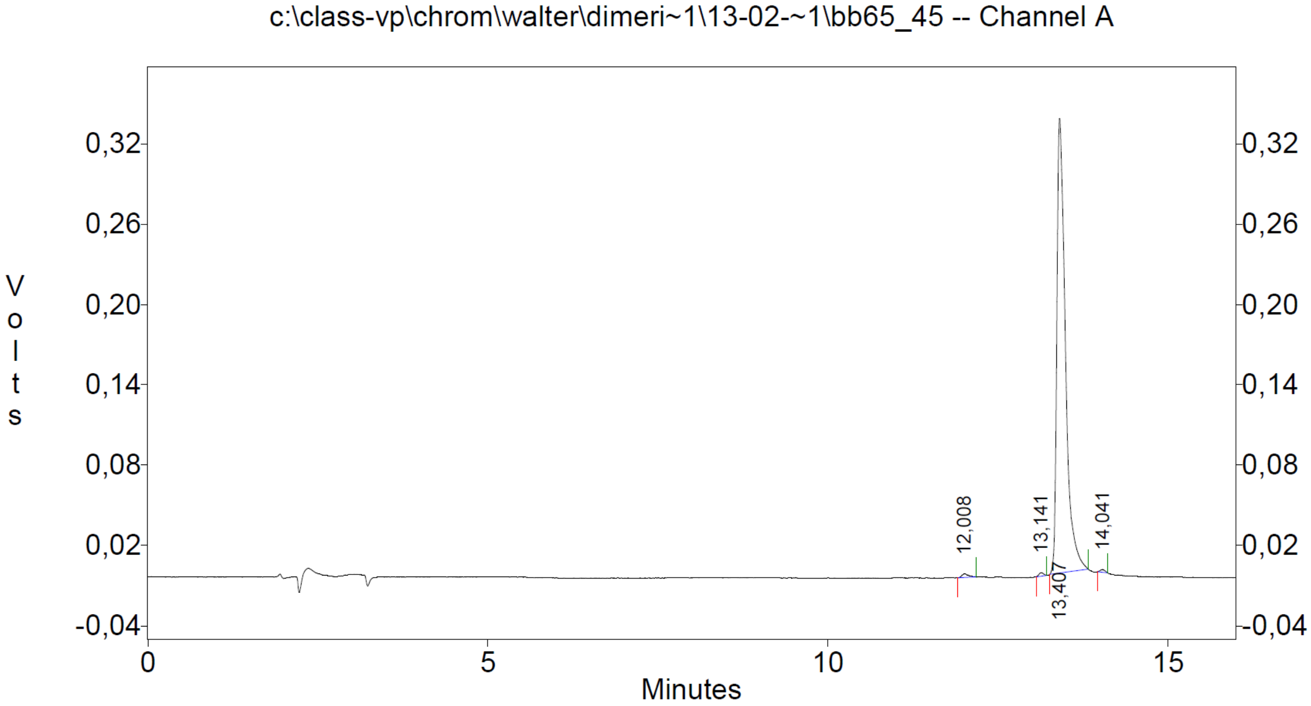

Linear gradient of MeCN in in 0.1 M ammonium acetate in H_2O (pH 7.0) from 5% to 95% over 20 min at a flow rate of 0.8 mL/min with detection at 288 nm.

Figure S3 RP-HPLC analysis of dimer **6ab**.

Channel A Results

Name	Time	Area	Area %	Conc (mg/L)
	13,10	20922	0,80	0,000
	13,48	2594441	99,20	0,000
Totals :		2615363	100,00	0,000

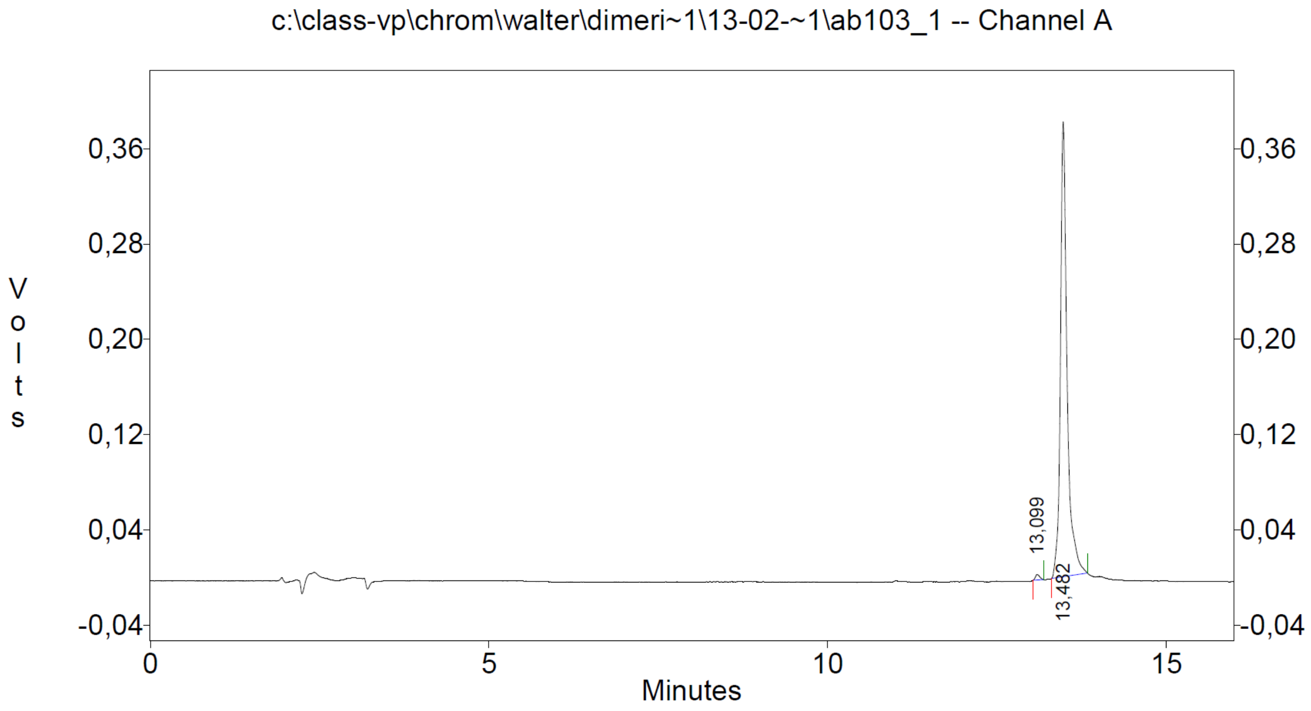

Linear gradient of MeCN in in 0.1 M ammonium acetate in H_2O (pH 7.0) from 5% to 95% over 20 min at a flow rate of 0.8 mL/min with detection at 288 nm.

Figure S4 ^{1}H NMR spectrum of 3,5,7,4''-tetra-*O*-isobutyryl-silybinB **2a** (400 MHz, $CDCl_3$)

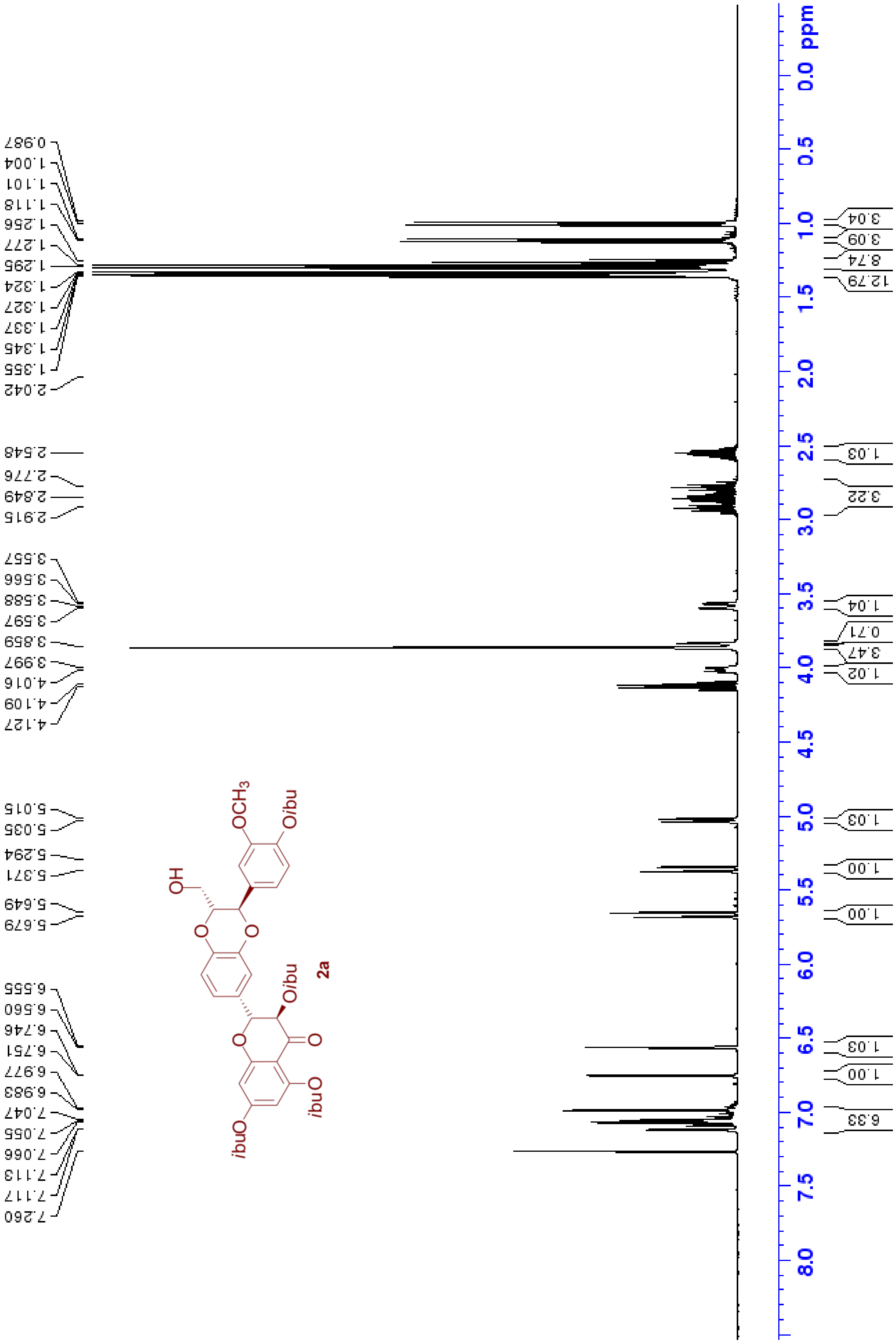

Figure S5 ^{13}C NMR spectrum of 3,5,7,4''-tetra-*O*-isobutyryl-silybinB **2a** (100 MHz, $CDCl_3$)

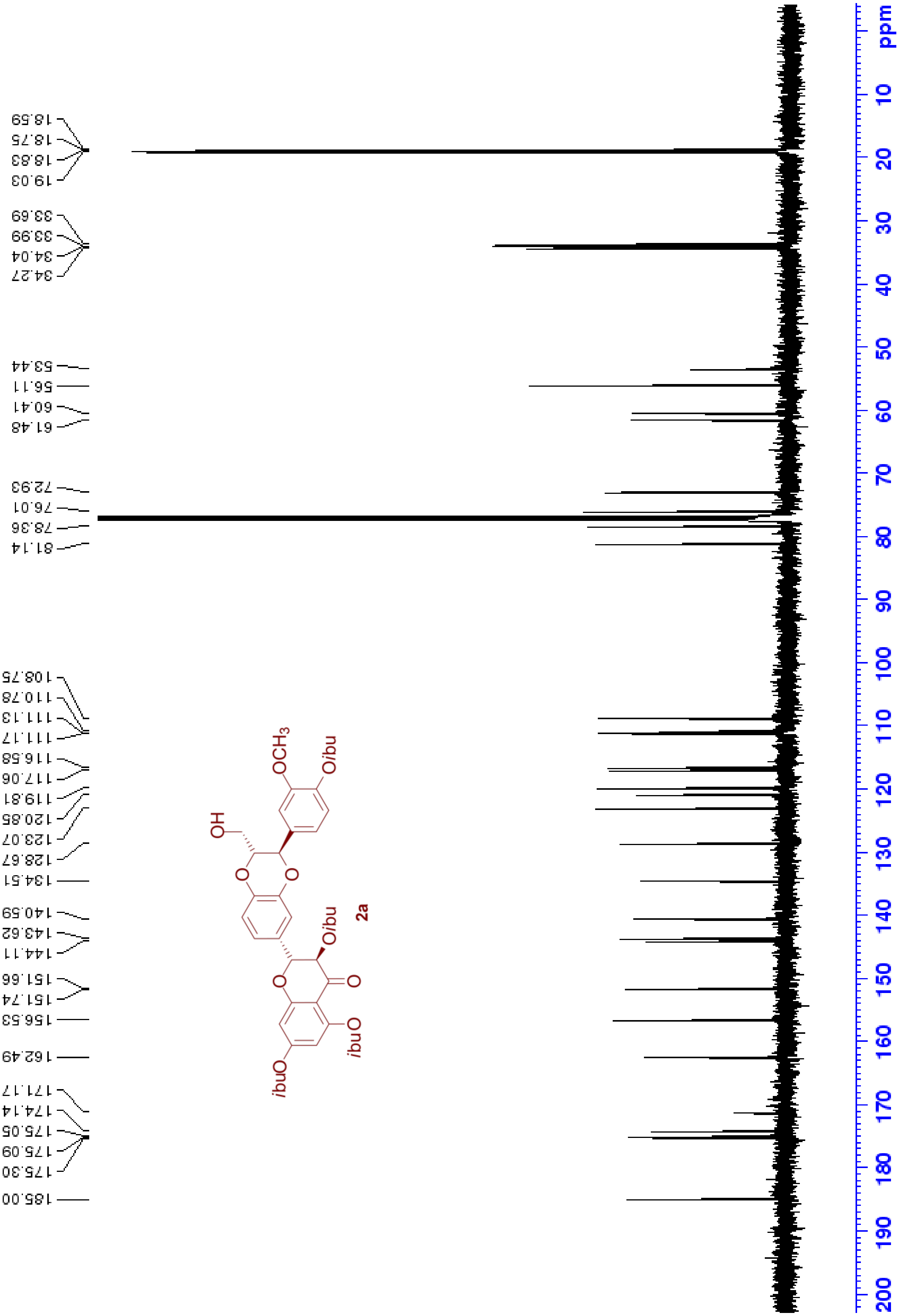

Figure S6 ^{1}H NMR spectrum of 3,5,7,4''-tetra-*O*-isobutyryl-silybinB **2b** (400 MHz, $CDCl_3$)

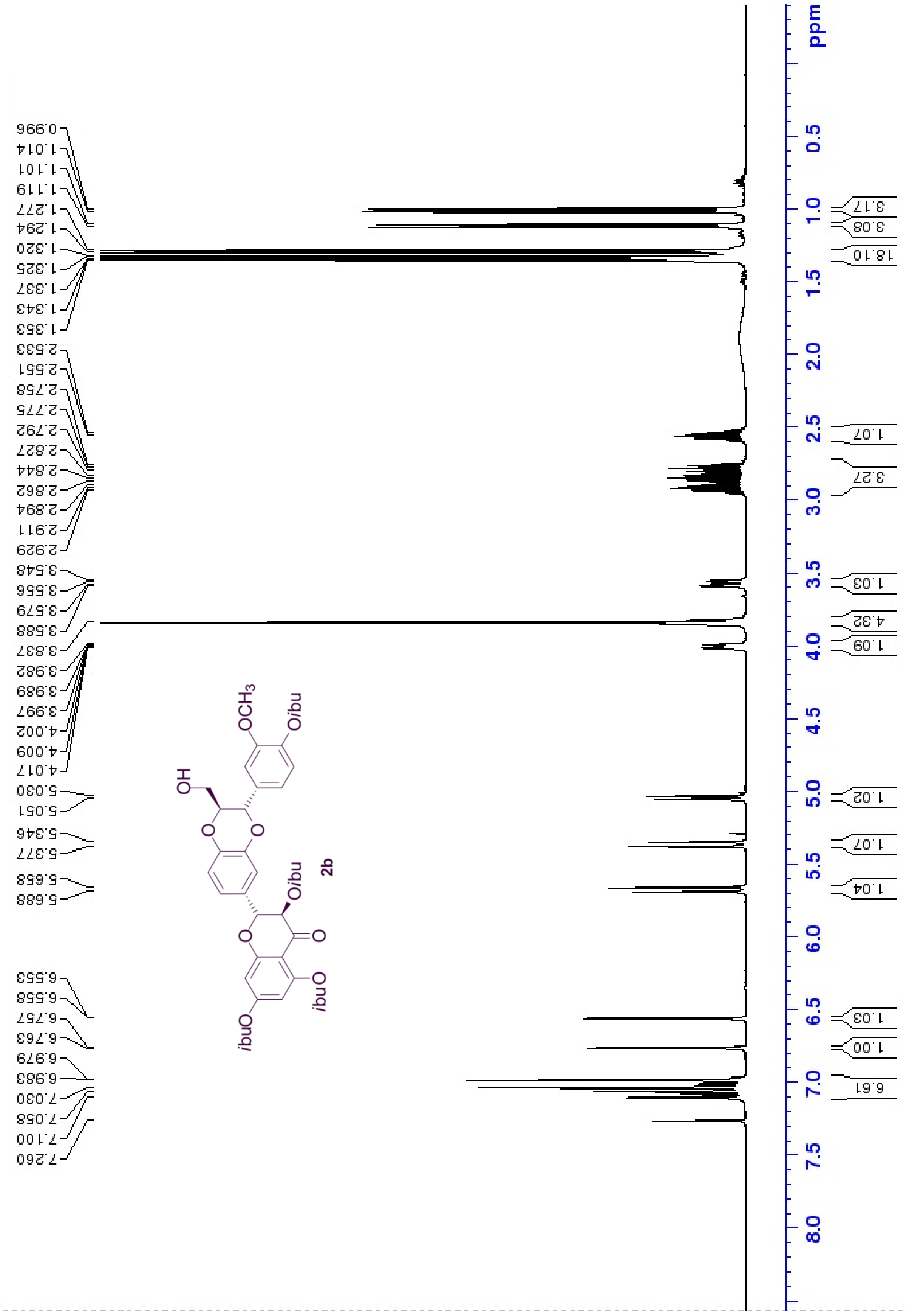

Figure S7 ^{13}C NMR spectrum of 3,5,7,4''-tetra-*O*-isobutyryl-silybinB **2b** (100 MHz, $CDCl_3$)

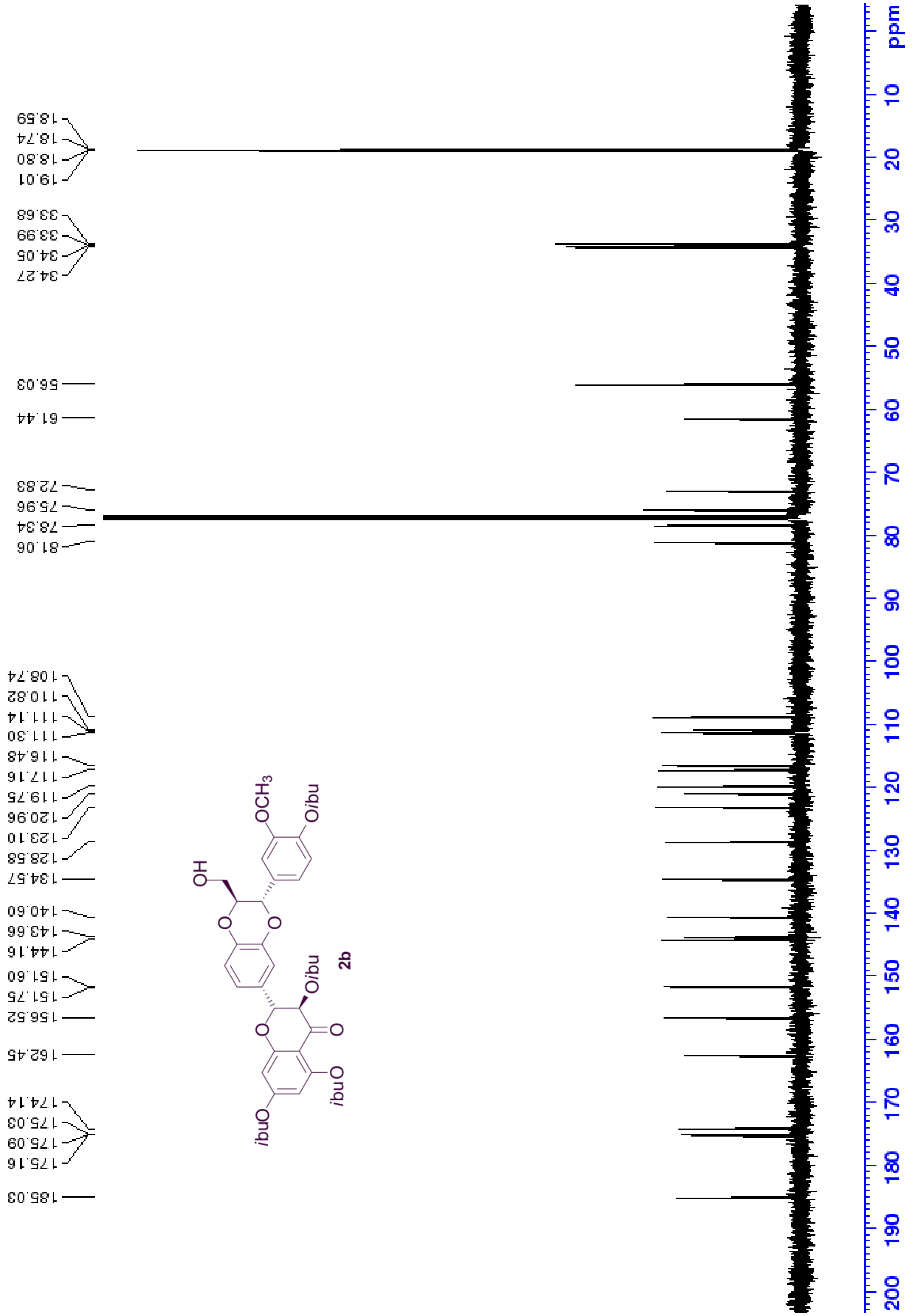

Figure S8 ^{1}H NMR spectrum of phosphoramidite derivative **4a** (400 MHz, $CDCl_3$)

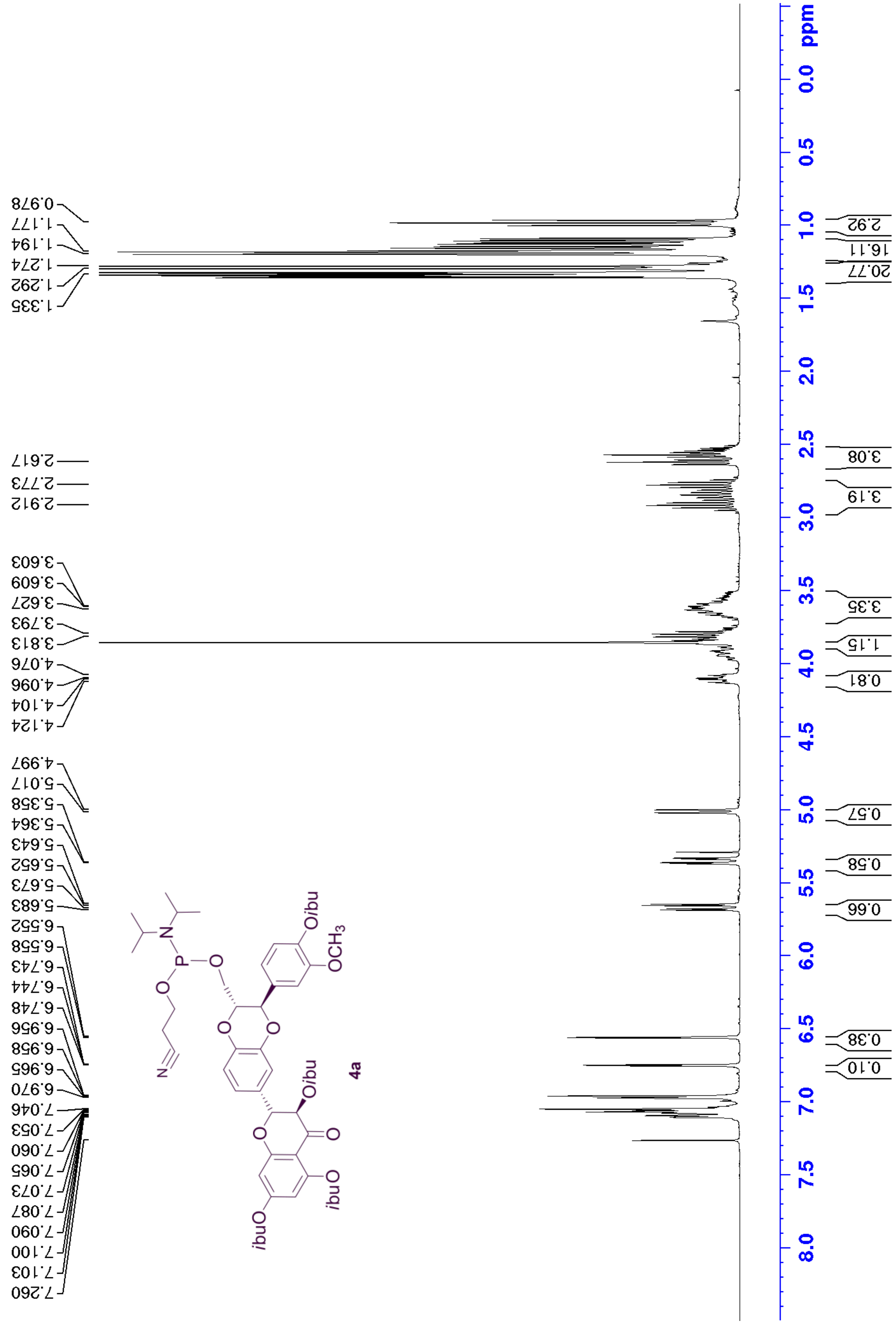

Figure S9 ^{13}C NMR spectrum of phosphoramidite derivative **4a** (100 MHz, $CDCl_3$)

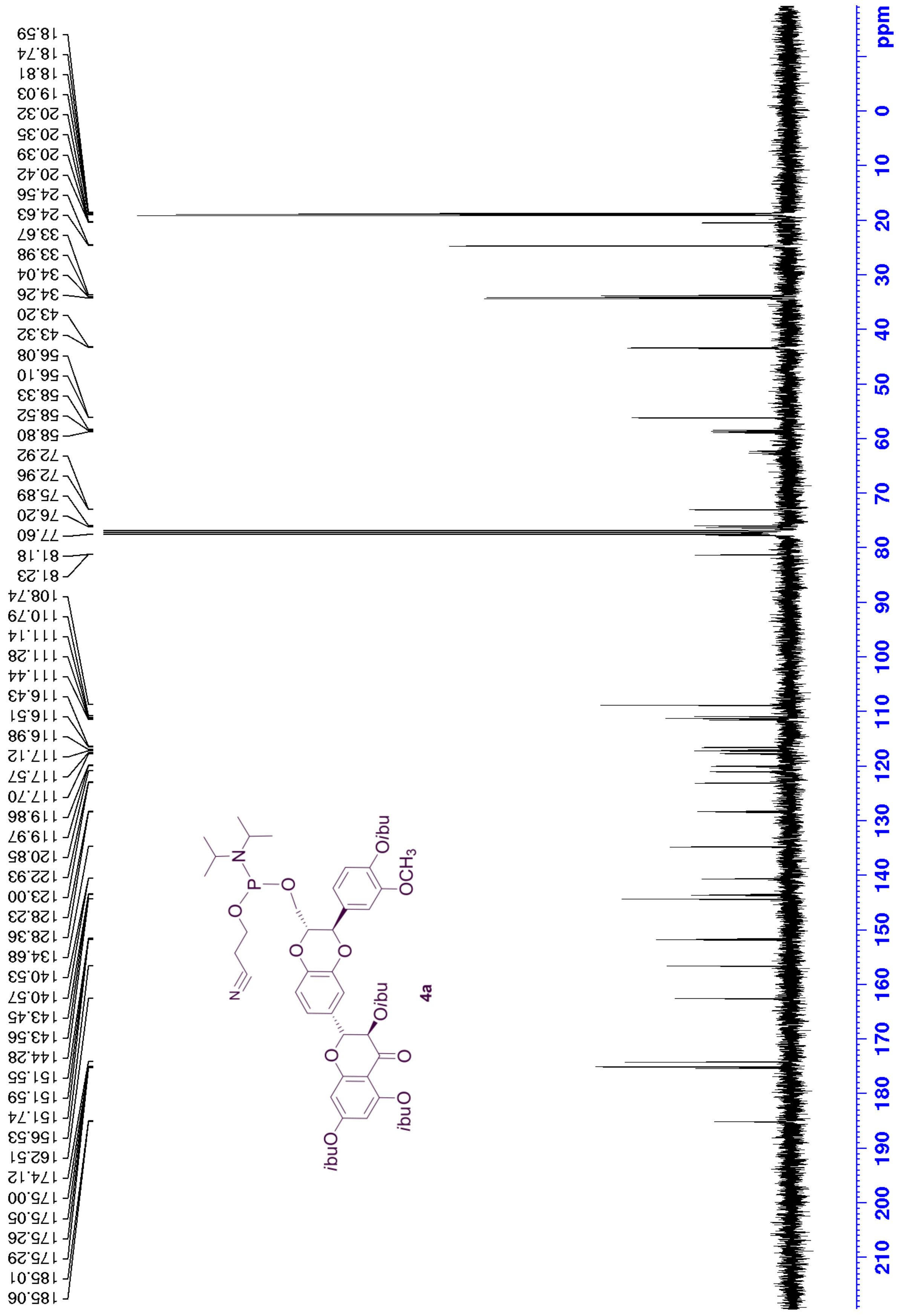

Figure S10 ^{31}P NMR spectrum of phosphoramidite derivative **4a** (168 MHz, $CDCl_3$)

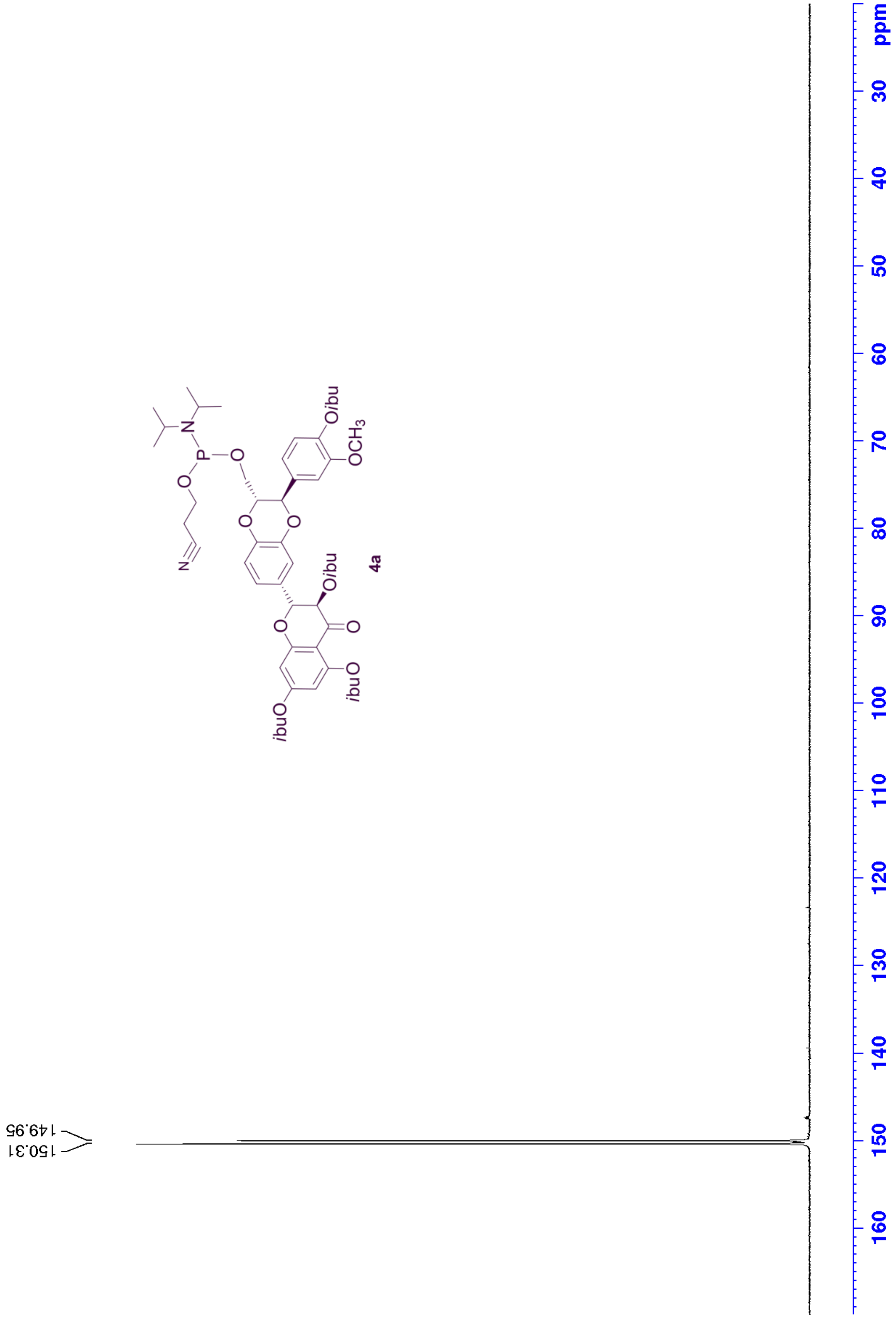

Figure S11 ^{1}H NMR spectrum of phosphoramidite derivative **4b** (400 MHz, $CDCl_3$)

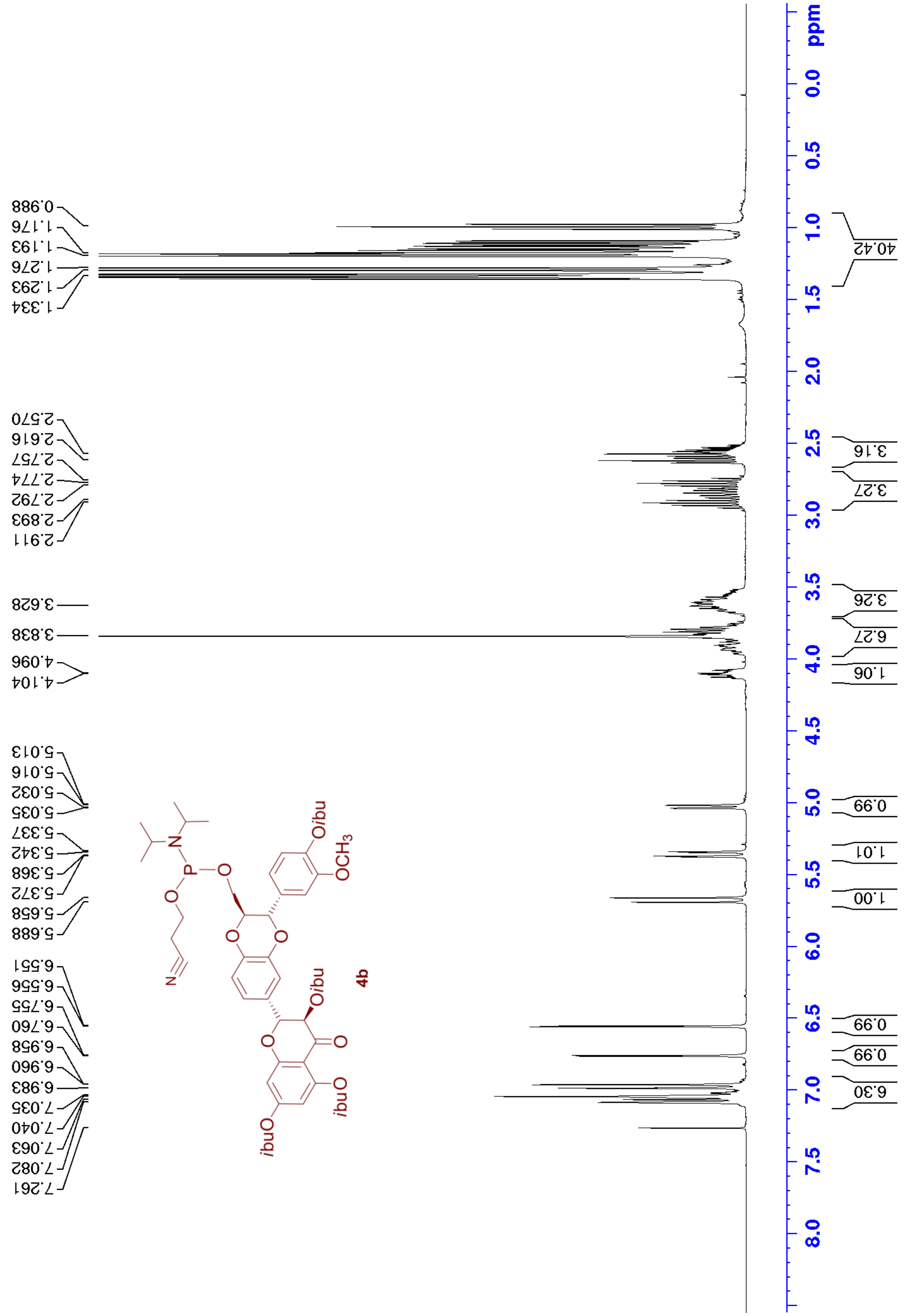

Figure S12 ^{13}C NMR spectrum of phosphoramidite derivative **4b** (400 MHz, $CDCl_3$)

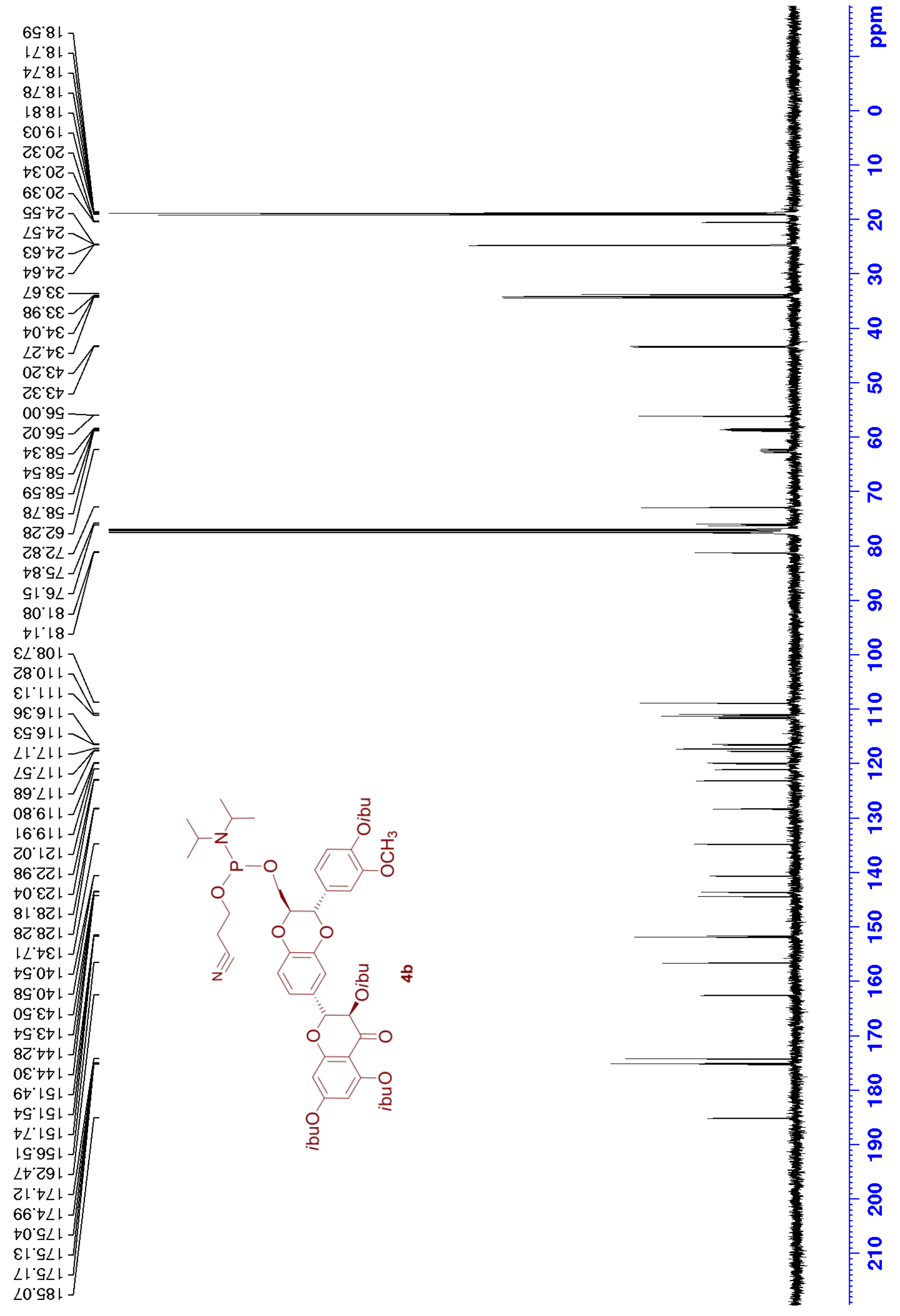

Figure S13 ^{31}P NMR spectrum of phosphoramidite derivative **4b** (168 MHz, $CDCl_3$)

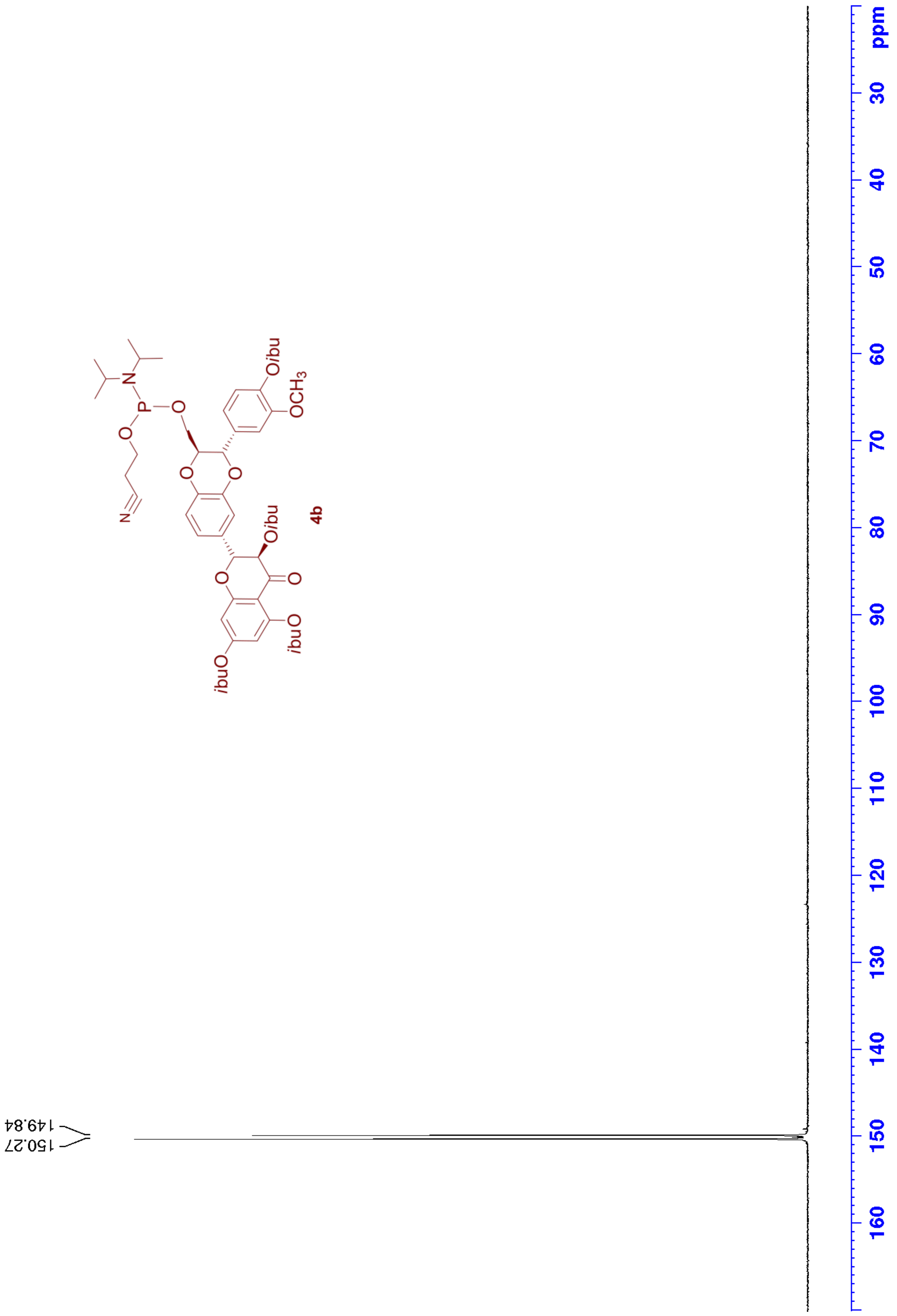

Figure S14 ^{1}H NMR spectrum of full protected dimer **5aa** (400 MHz, $CDCl_3$)

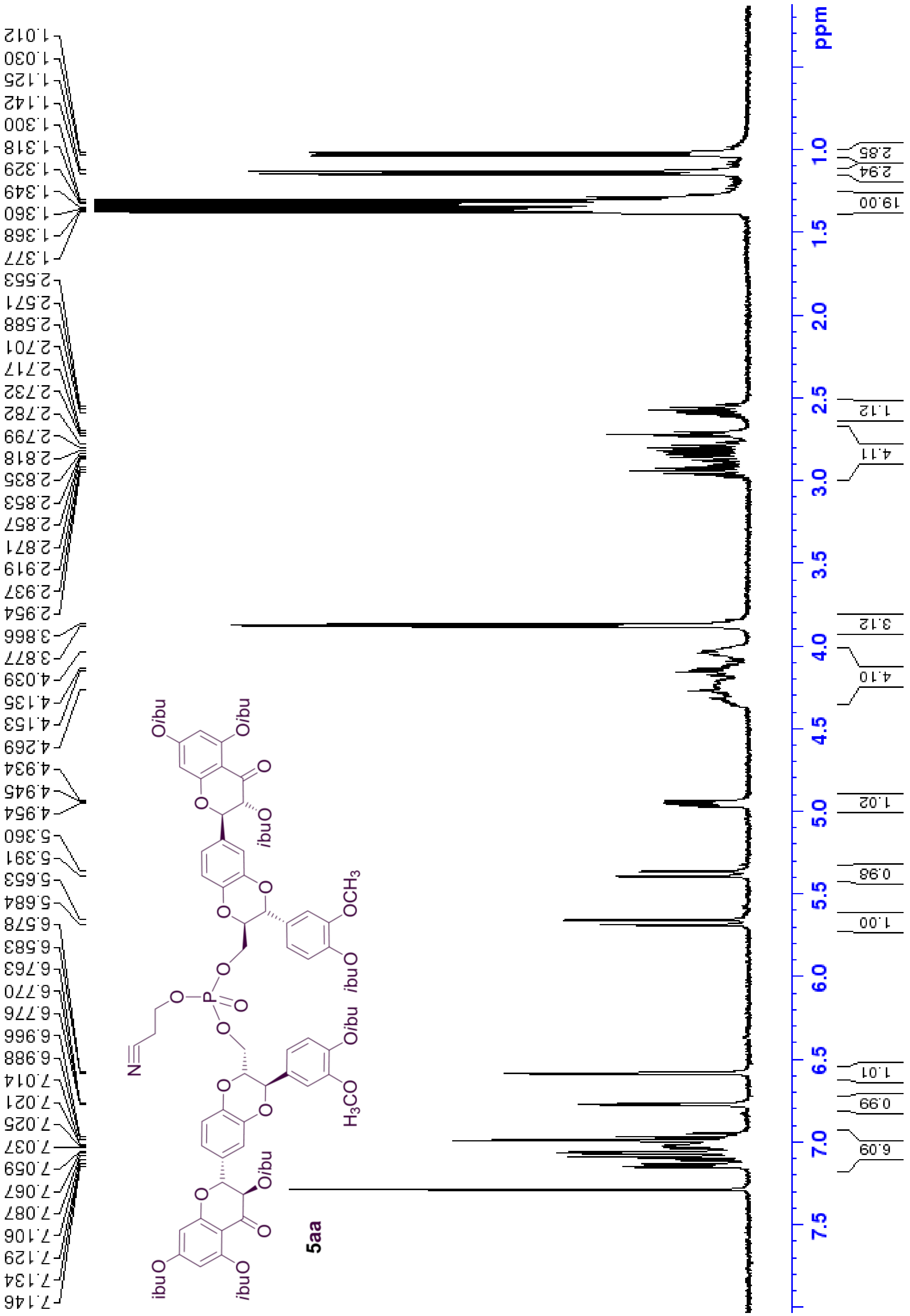

Figure S15 ^{13}C NMR spectrum of full protected dimer **5aa** (100 MHz, $CDCl_3$)

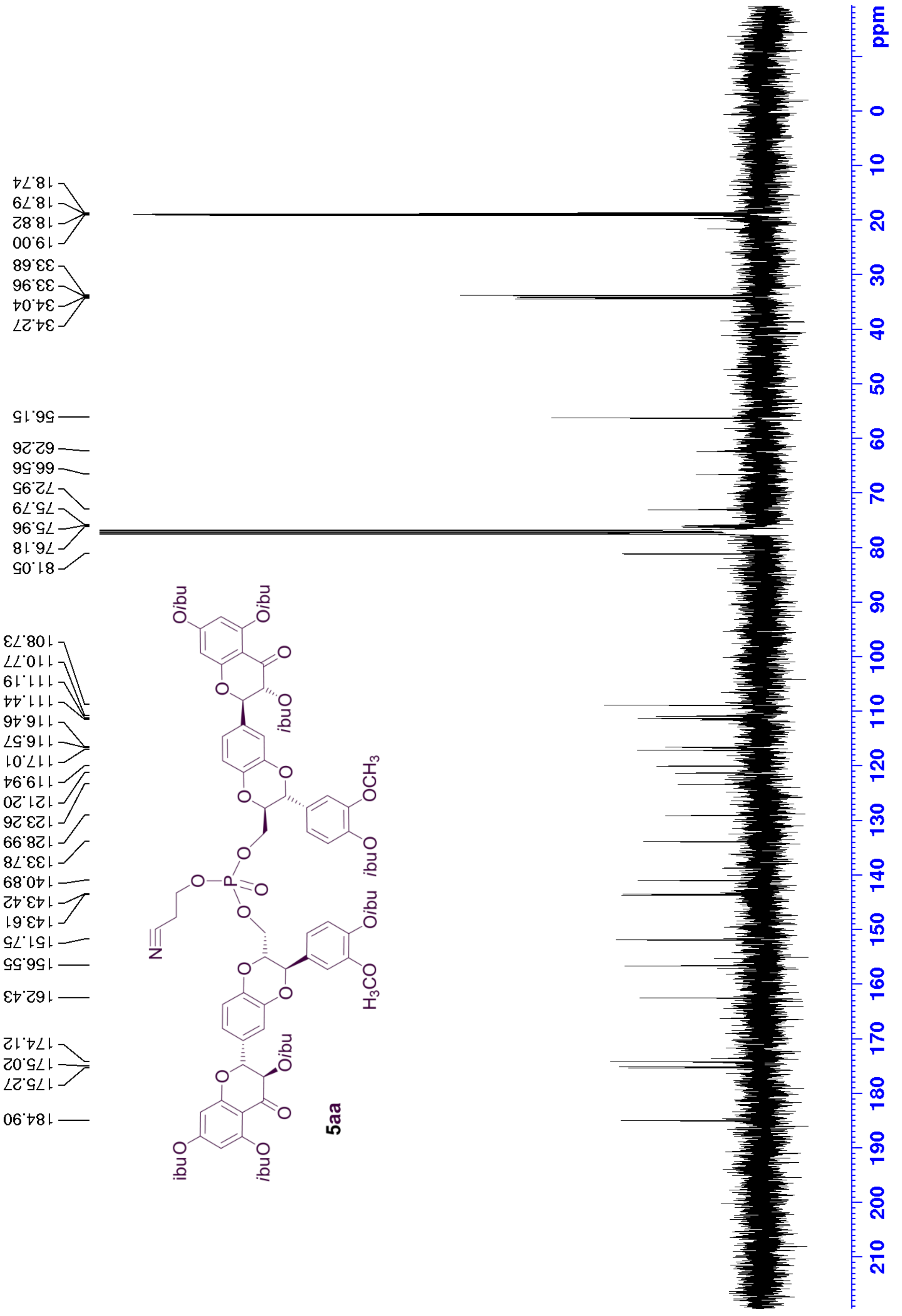

Figure S16 ^{31}P NMR spectrum of full protected dimer **5aa** (168 MHz, $CDCl_3$)

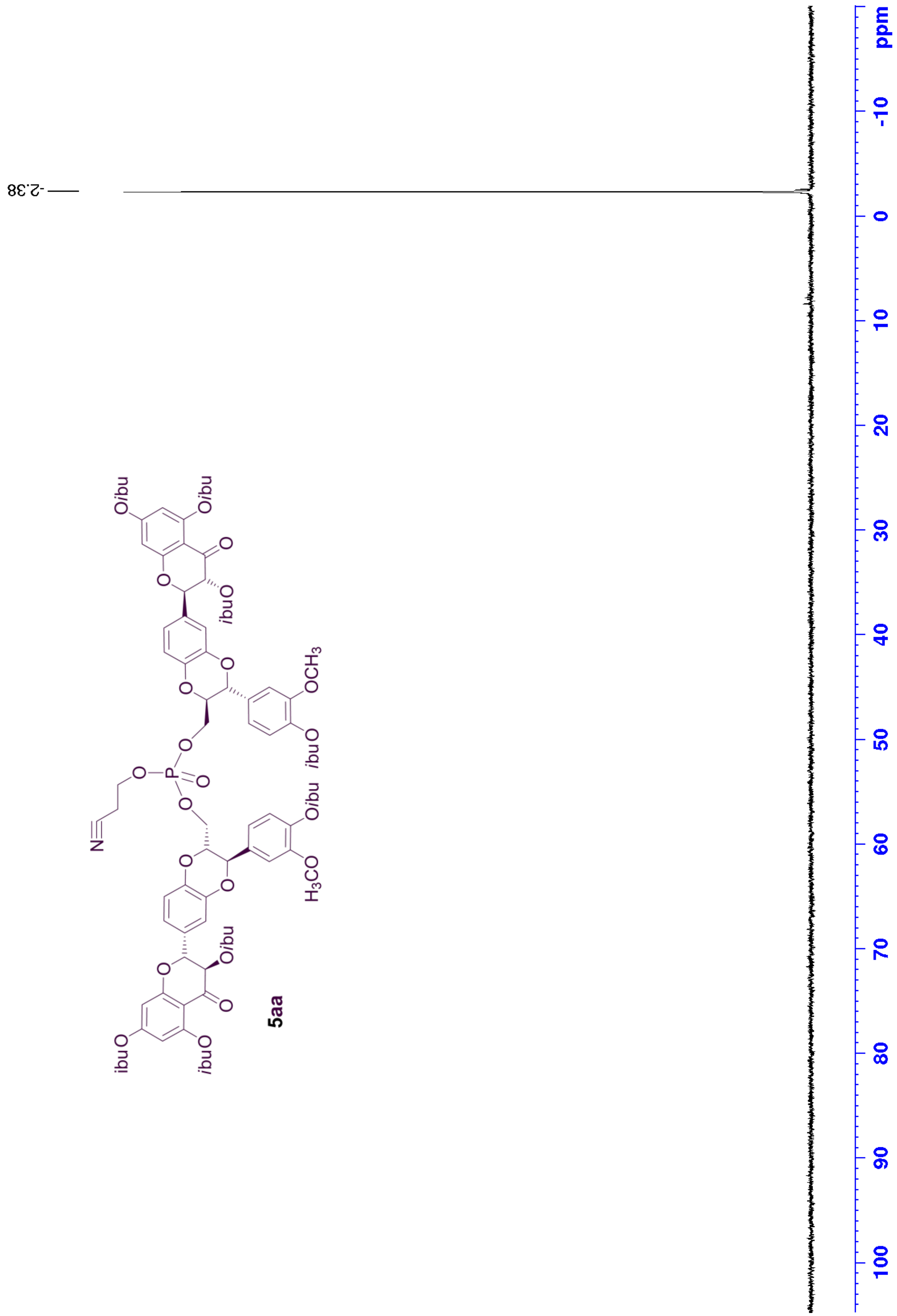

Figure S17 ^{1}H NMR spectrum of full protected dimer **5bb** (400 MHz, $CDCl_3$)

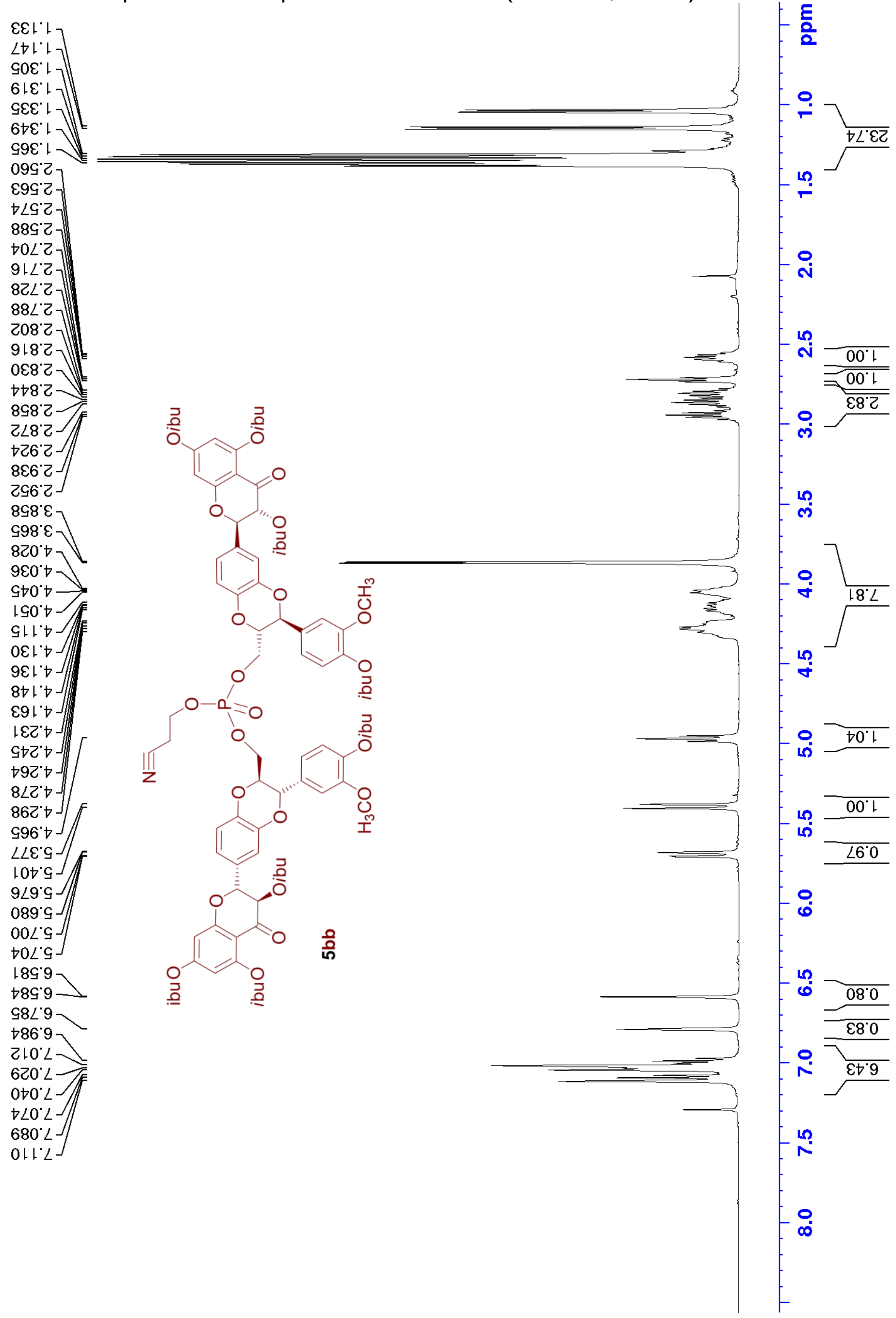

Figure S18 ^{13}C NMR spectrum of full protected dimer **5bb** (125 MHz, $CDCl_3$)

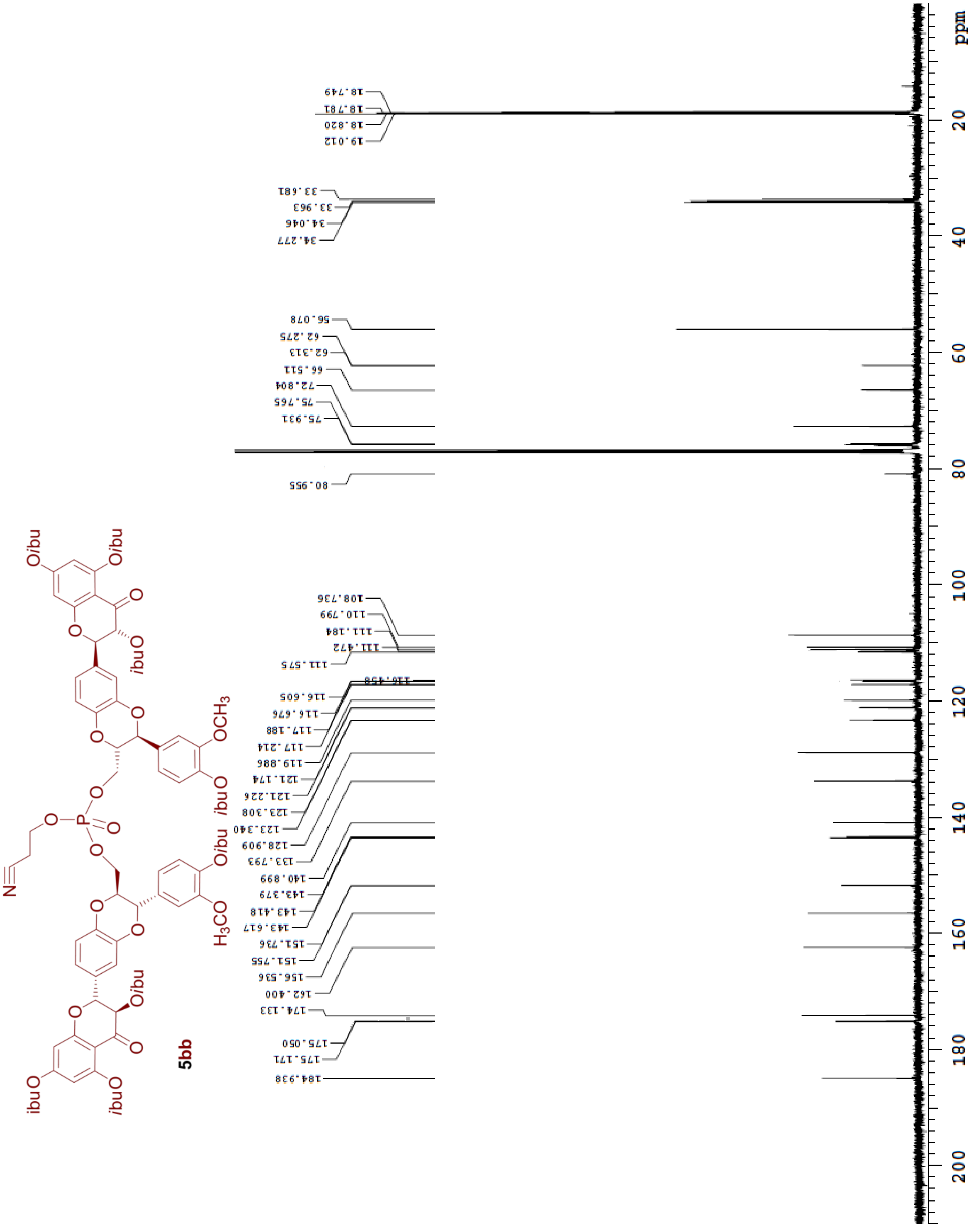

Figure S19 ^{31}P NMR spectrum of full protected dimer **5bb** (168 MHz, CDCl$_3$)

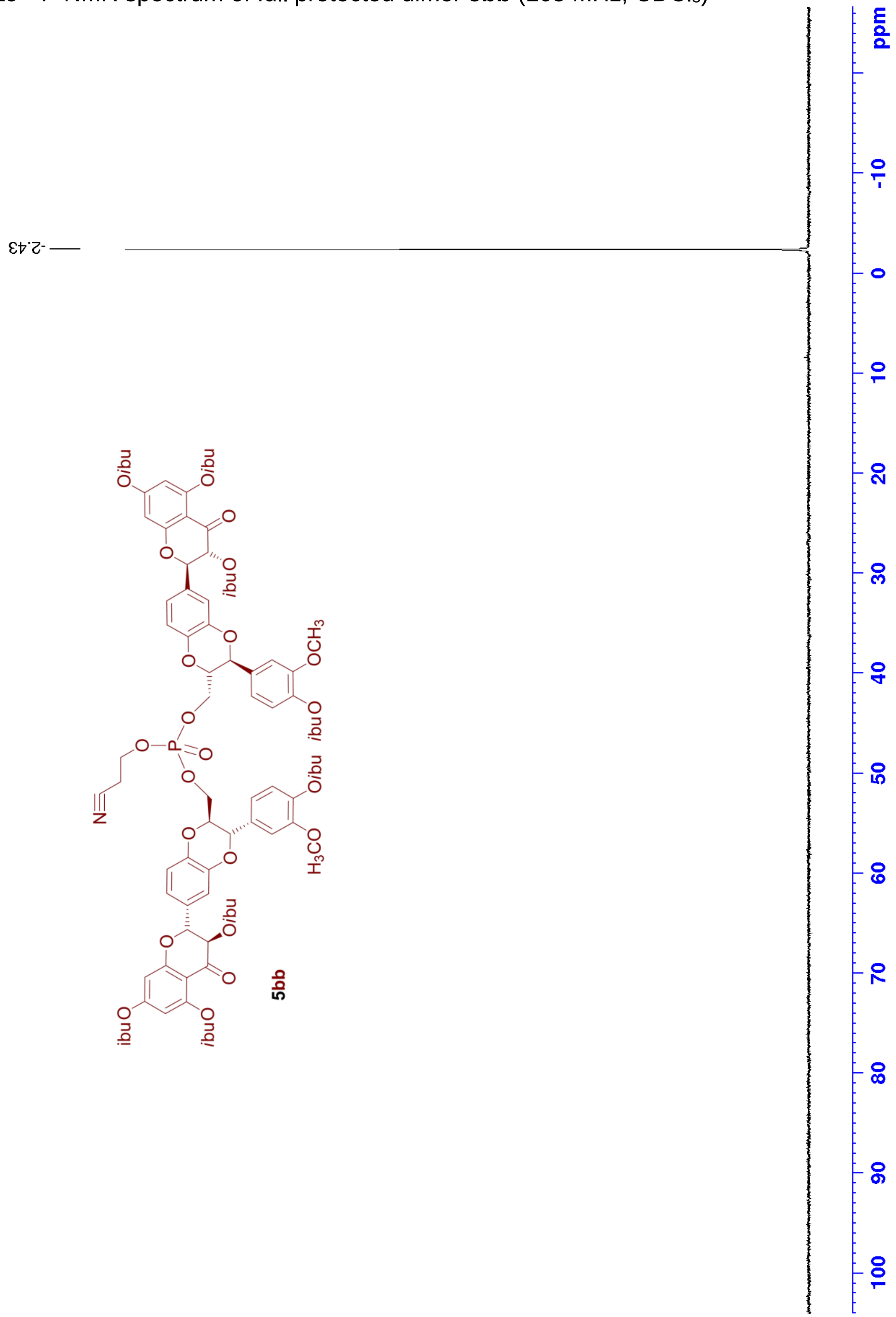

Figure S20 ^{1}H NMR spectrum of full protected dimer **5ab** (400 MHz, $CDCl_3$)

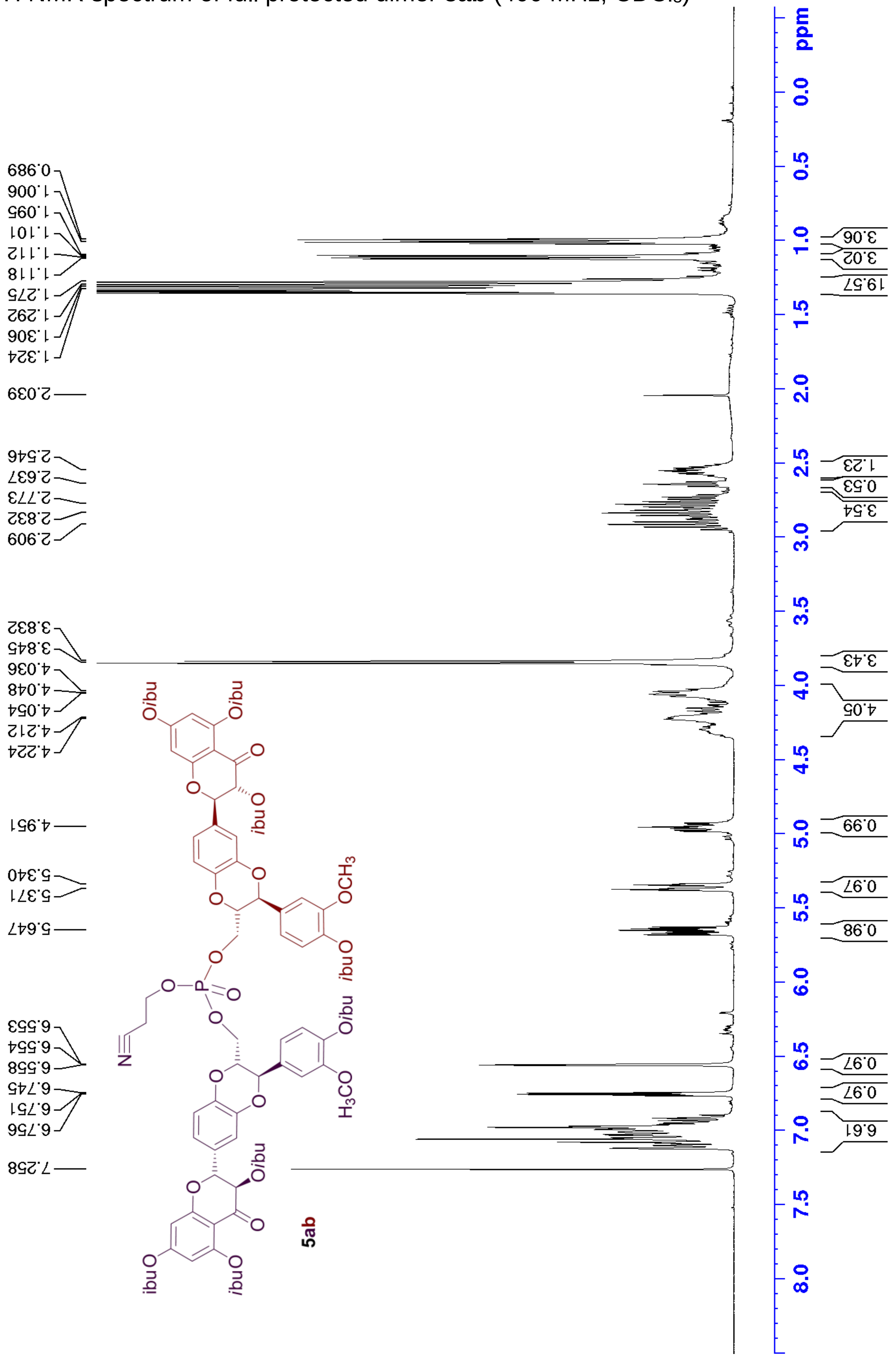

Figure S21 ^{13}C NMR spectrum of full protected dimer **5ab** (100 MHz, $CDCl_3$)

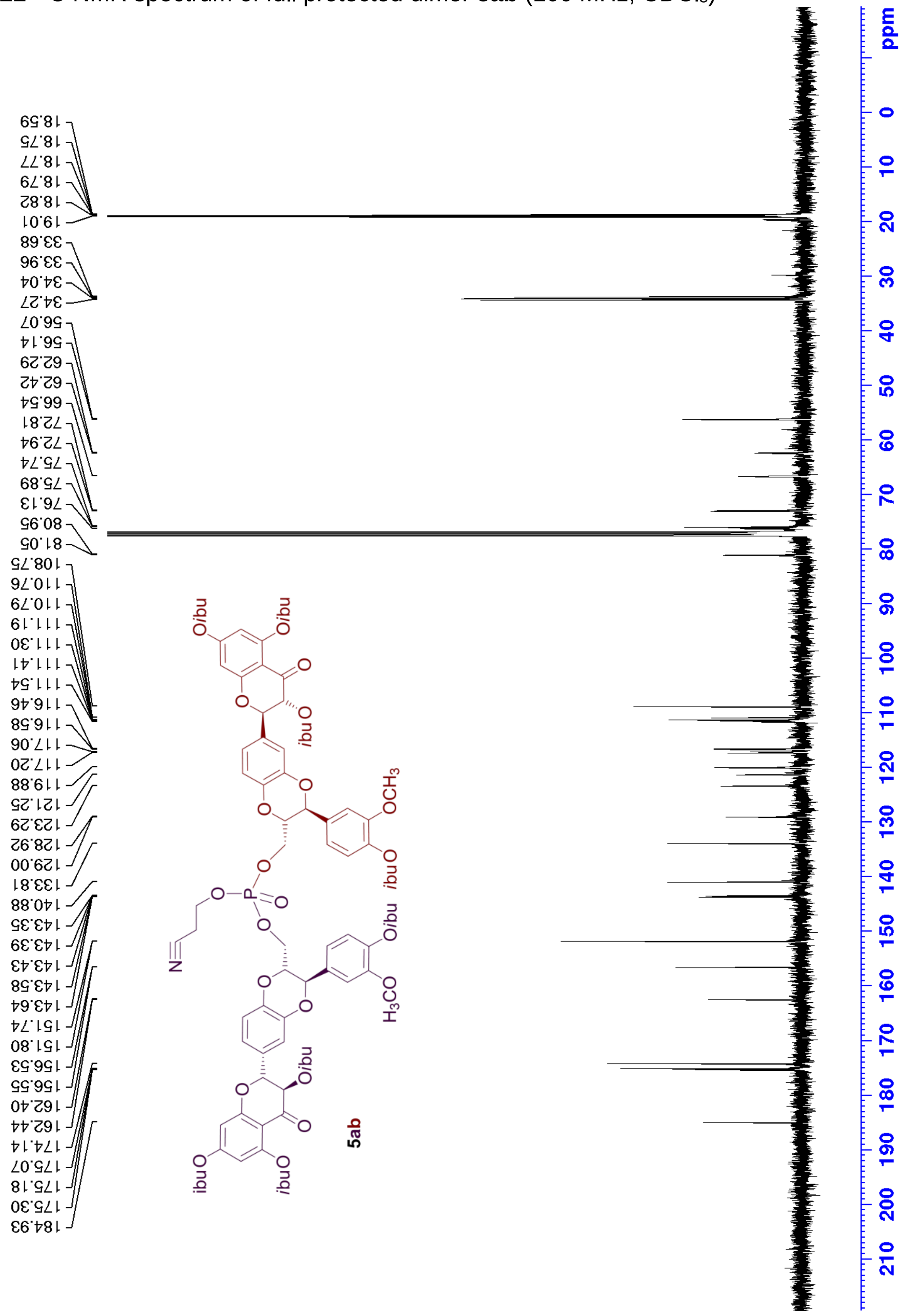

Figure S22 ^{31}P NMR spectrum of full protected dimer **5ab** (168 MHz, $CDCl_3$)

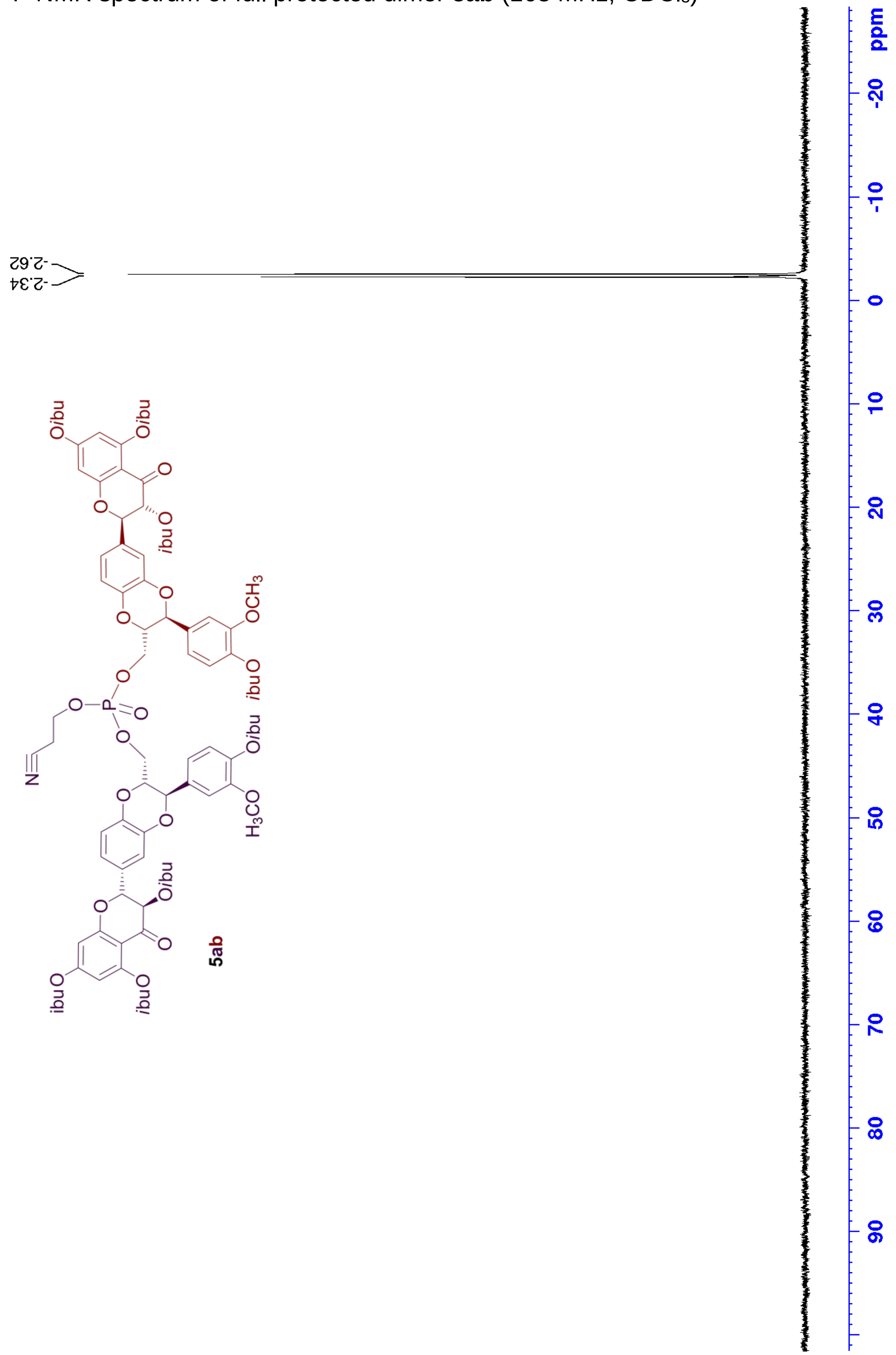

Figure S23 ^{1}H NMR spectrum of dimer **6aa** (400 MHz, DMSO-d$_6$)

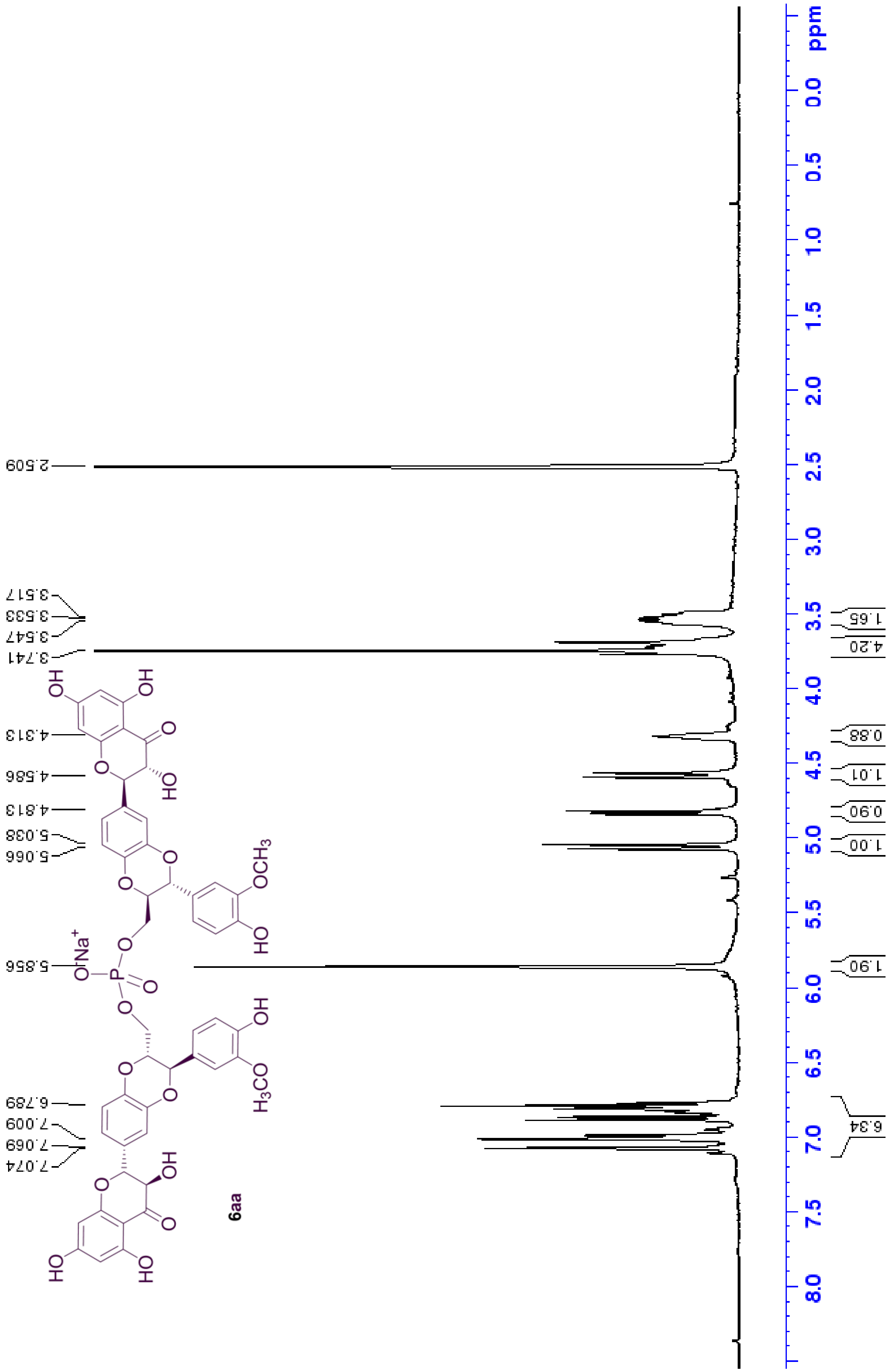

Figure S24 ^{13}C NMR ATP spectrum of dimer **6aa** (100 MHz, DMSO-d$_6$)

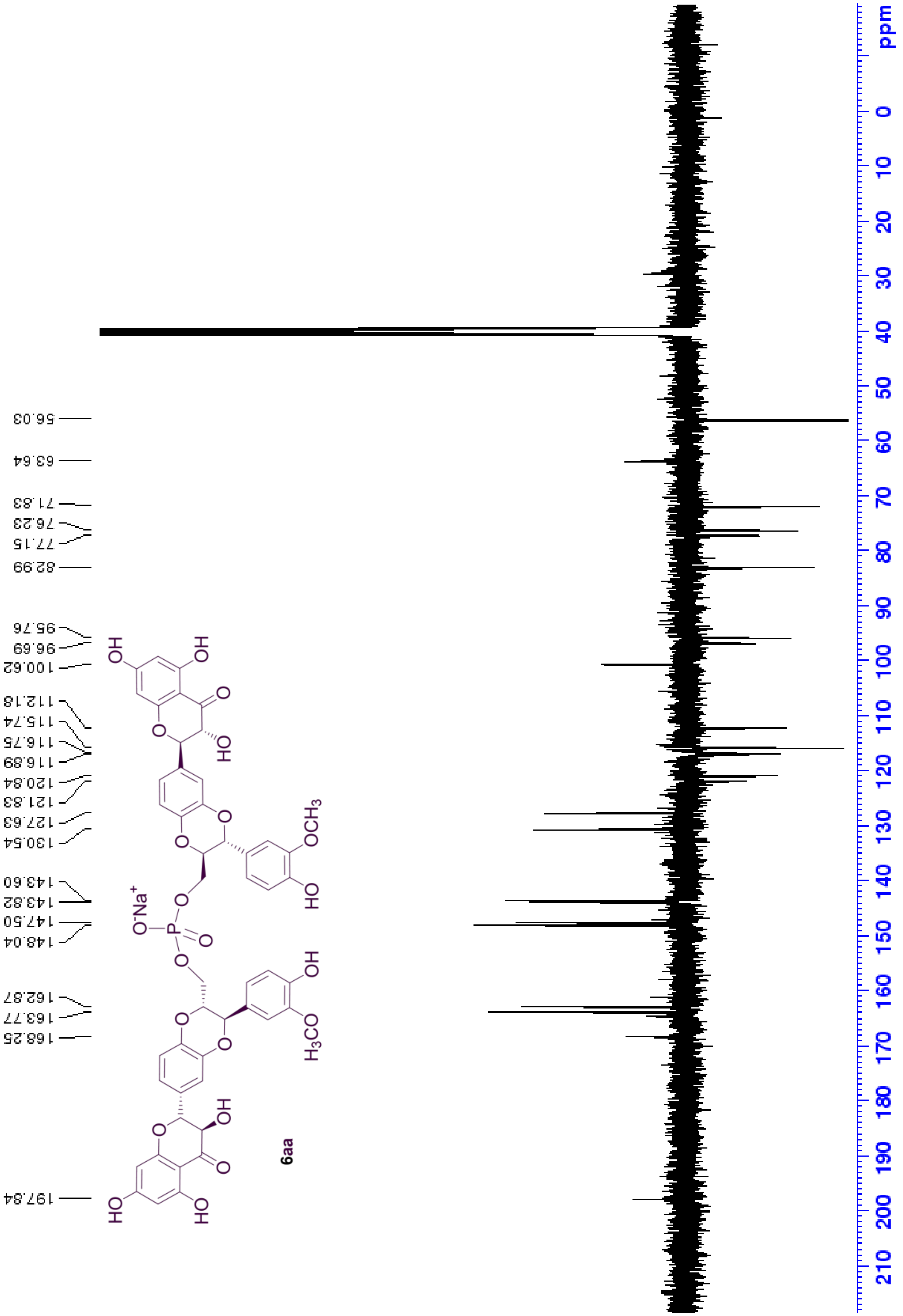

Figure S25 ^{31}P NMR spectrum of dimer **6aa** (168 MHz, DMSO-d$_6$)

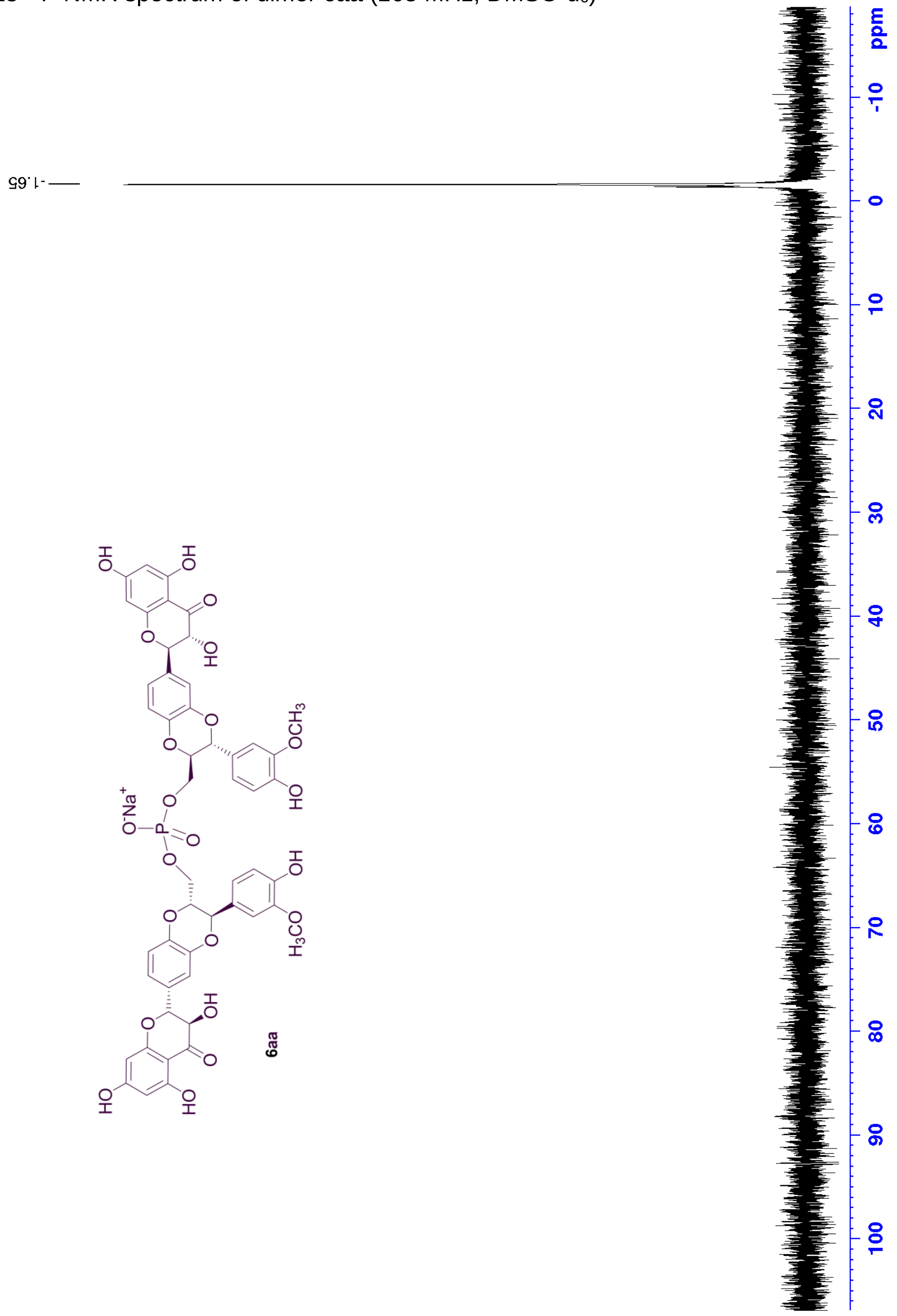

Figure S26 ^{1}H NMR spectrum of dimer **6bb** (400 MHz, DMSO-d6)

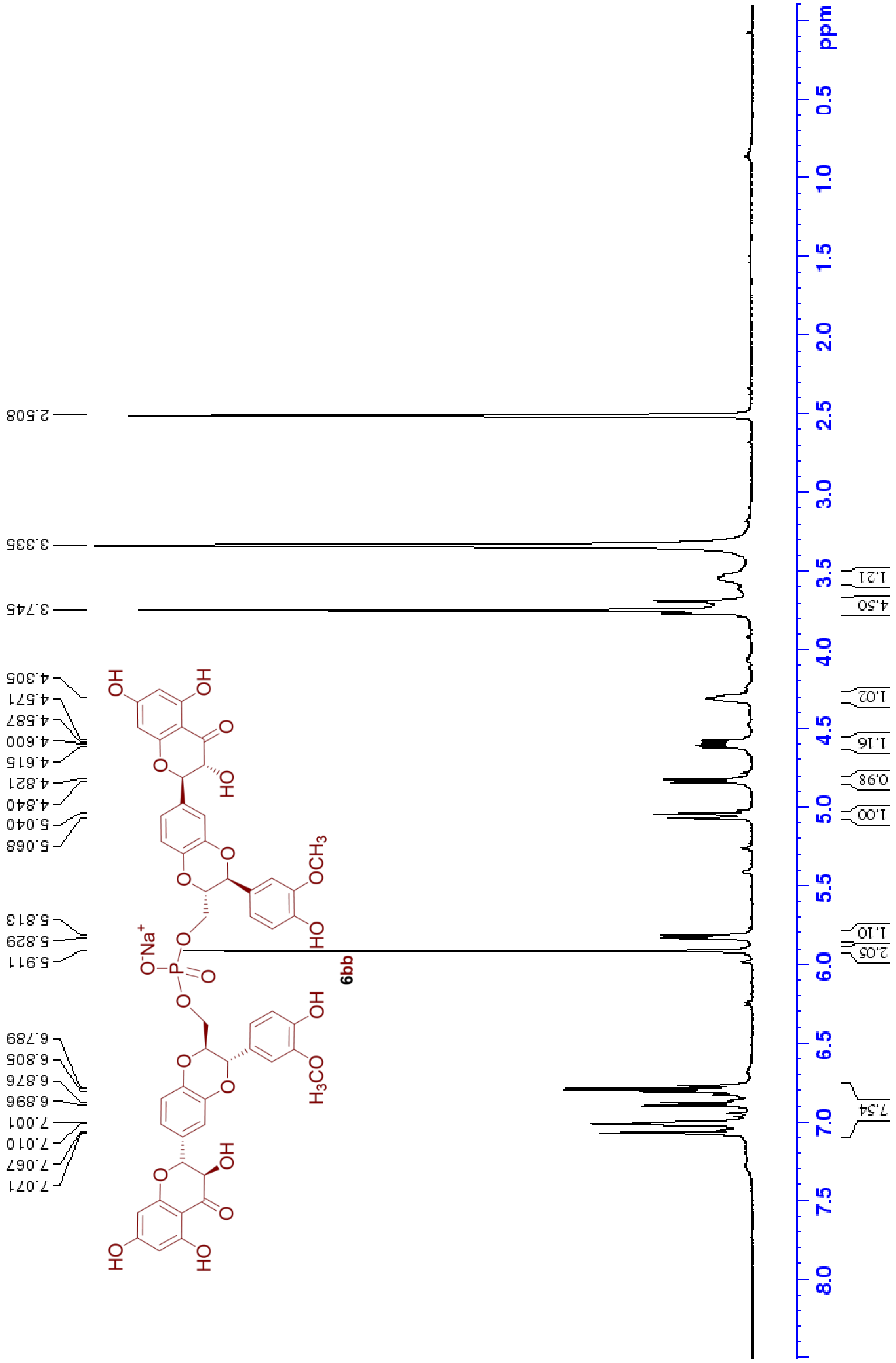

Figure S27 ^{13}C NMR ATP spectrum of dimer **6bb** (100 MHz, DMSO-d_6)

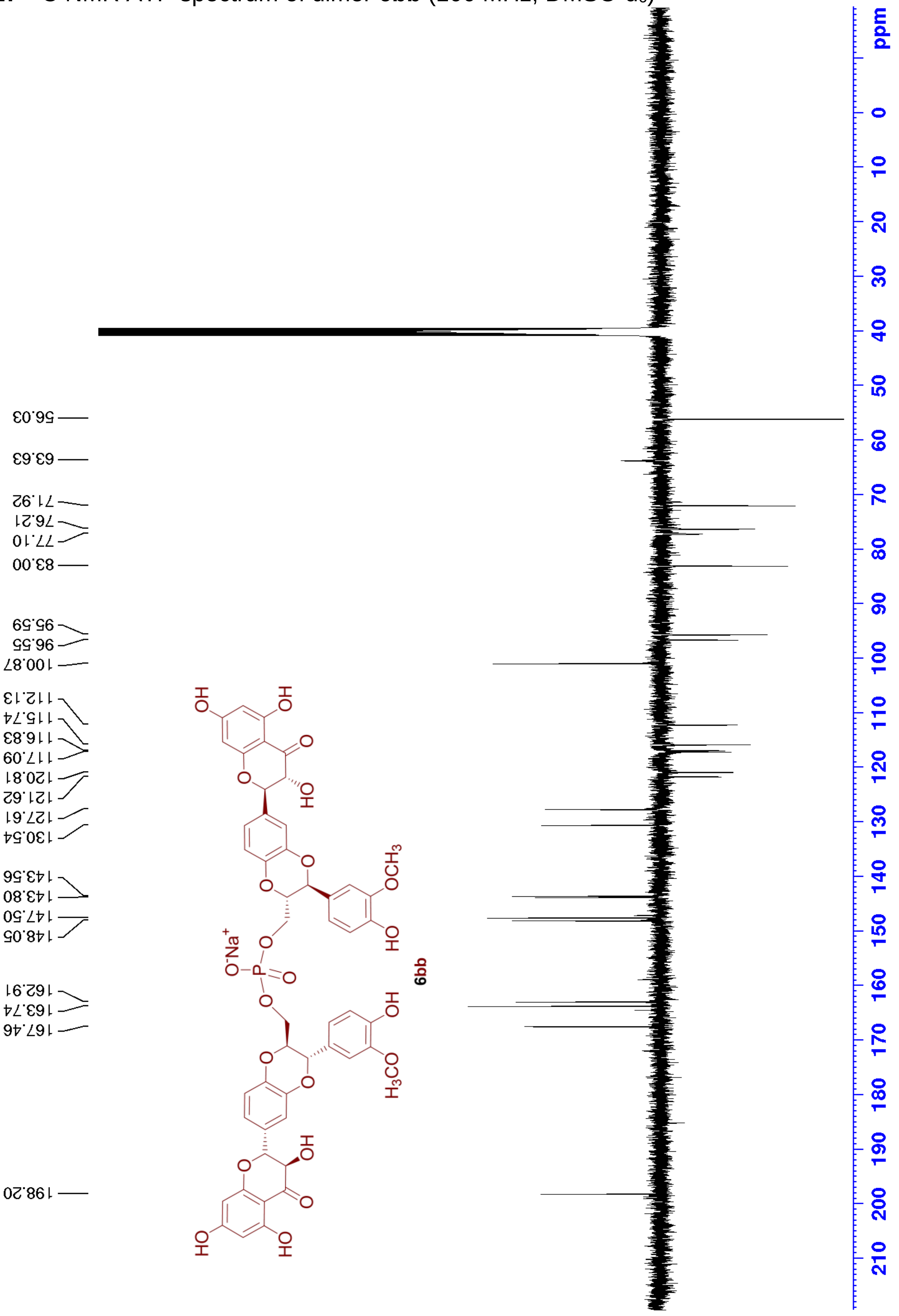

Figure S28 ^{31}P NMR spectrum of dimer **6bb** (168 MHz, DMSO-d$_6$)

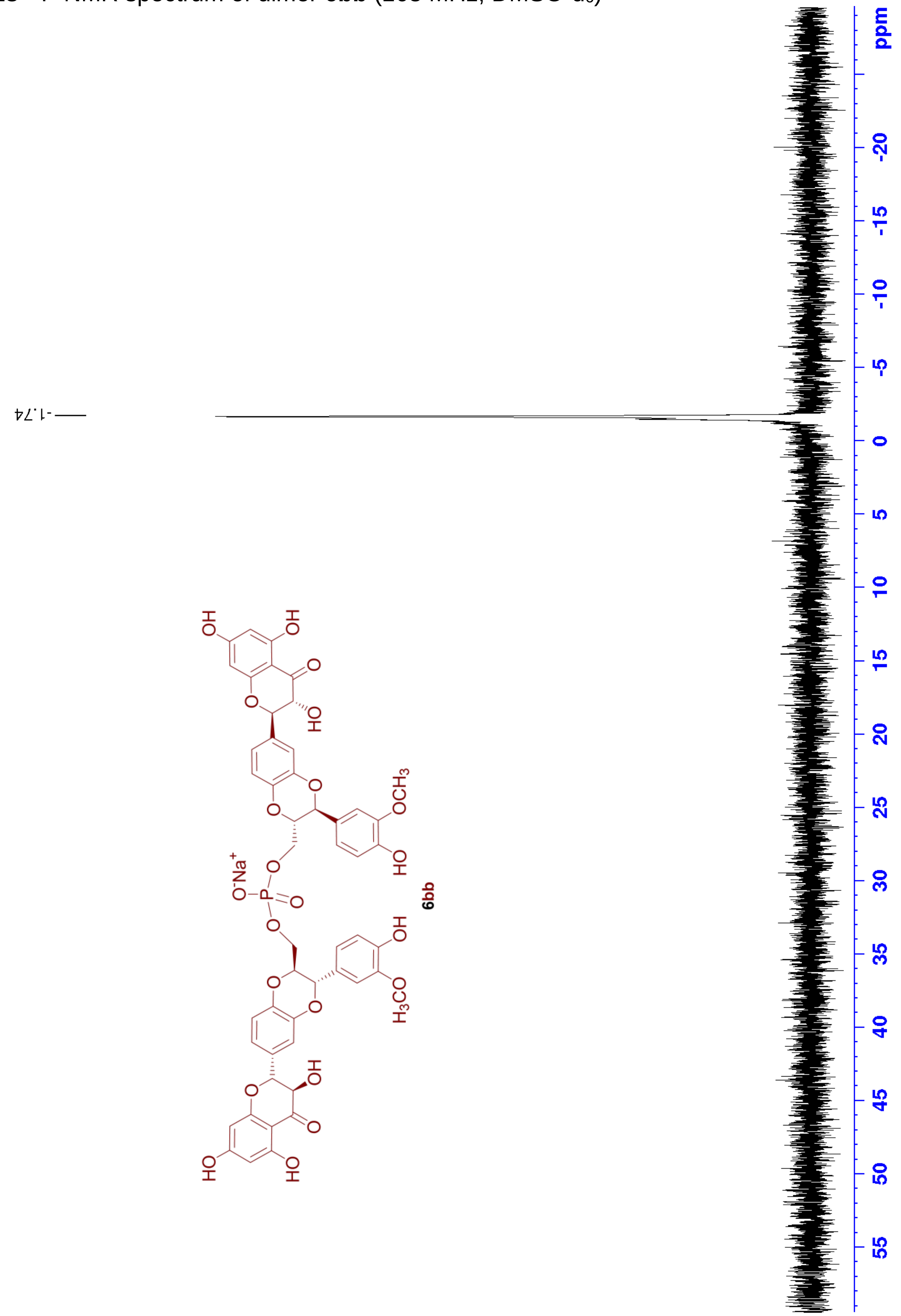

Figure S29 ^{1}H NMR spectrum of dimer **6ab** (400 MHz, CD_3OD)

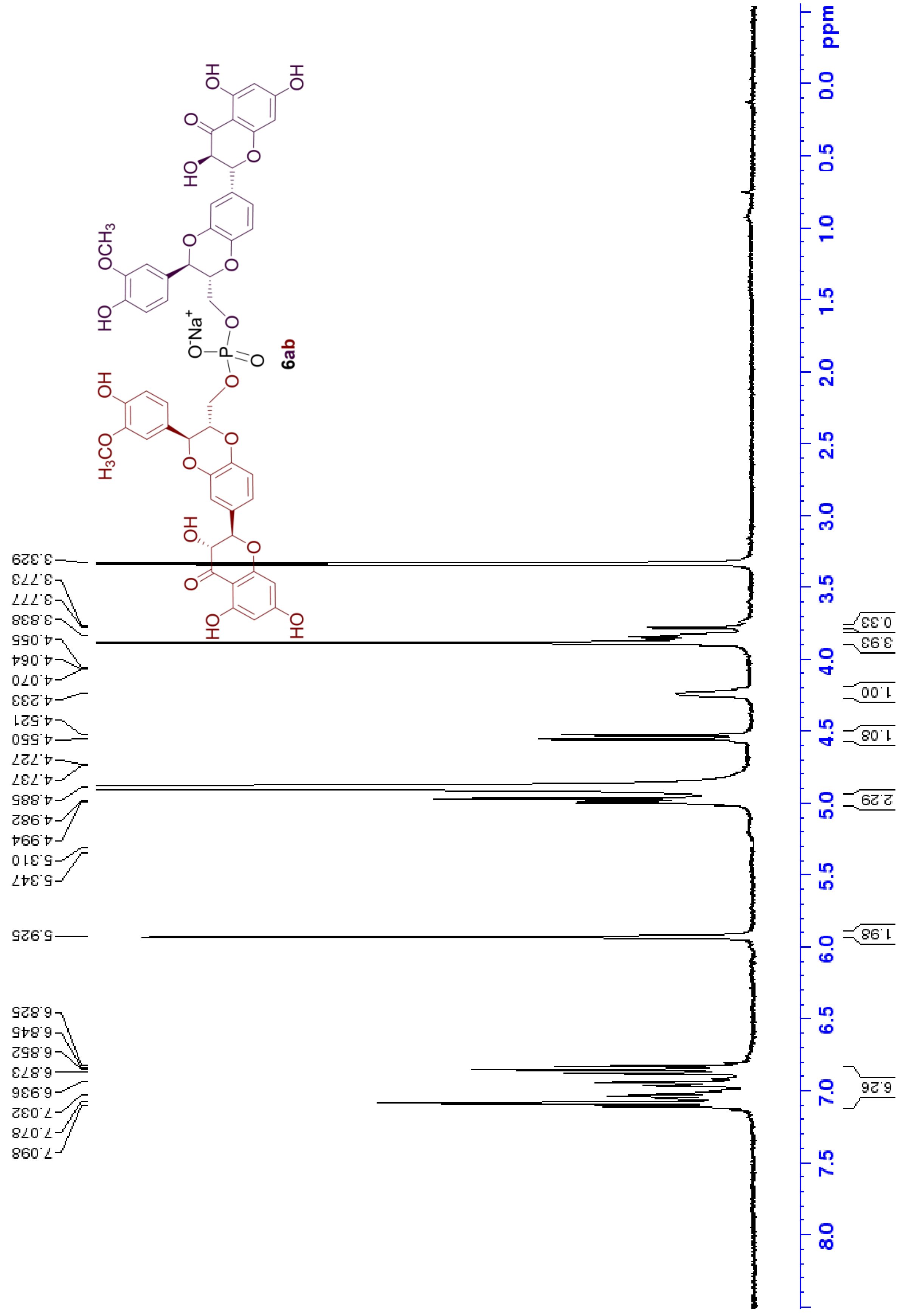

Figure S30 ^{13}C NMR spectrum of dimer **6ab** (100 MHz, CD_3OD)

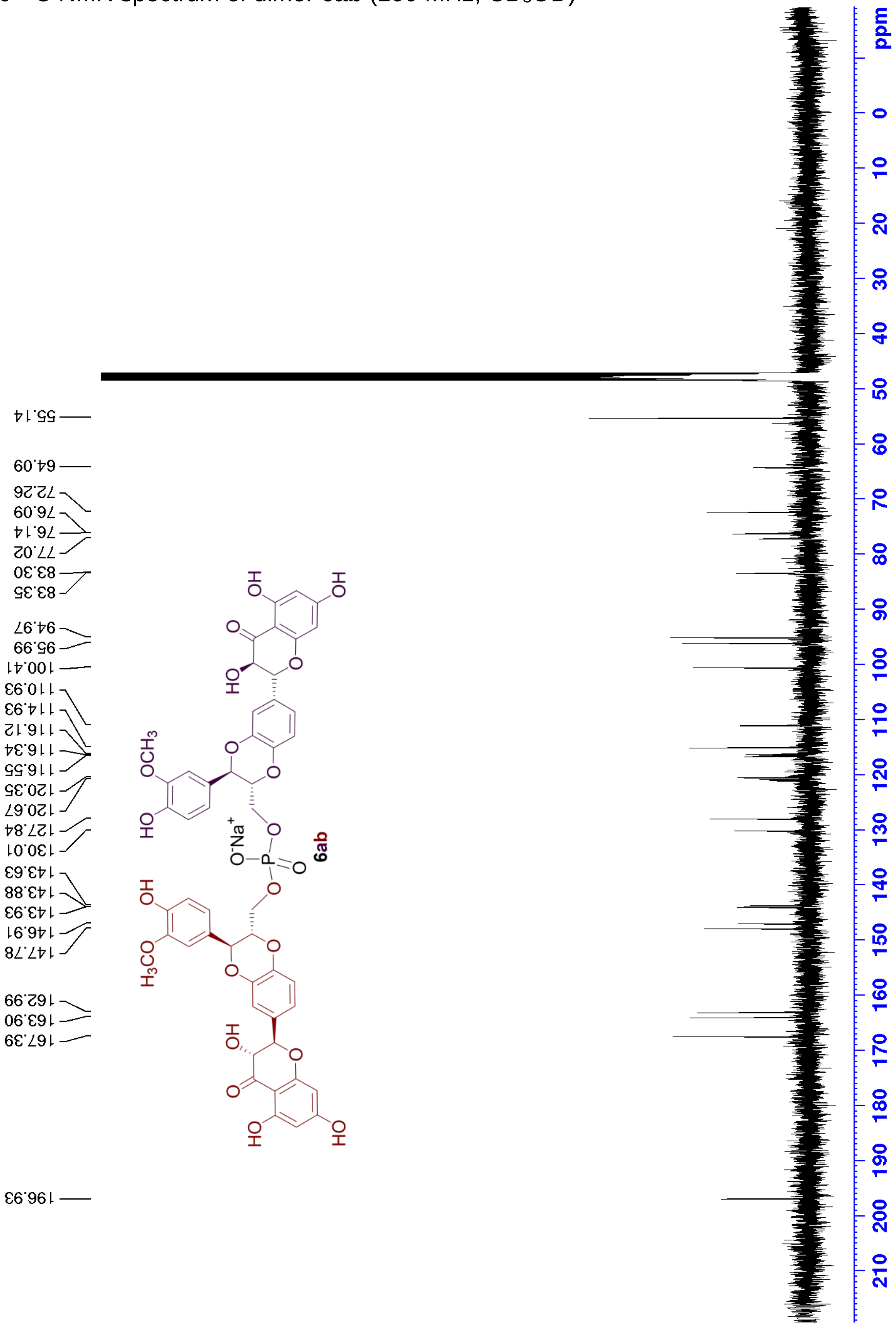

Figure S31 ^{31}P NMR spectrum of dimer **6ab** (168 MHz, CD_3OD)

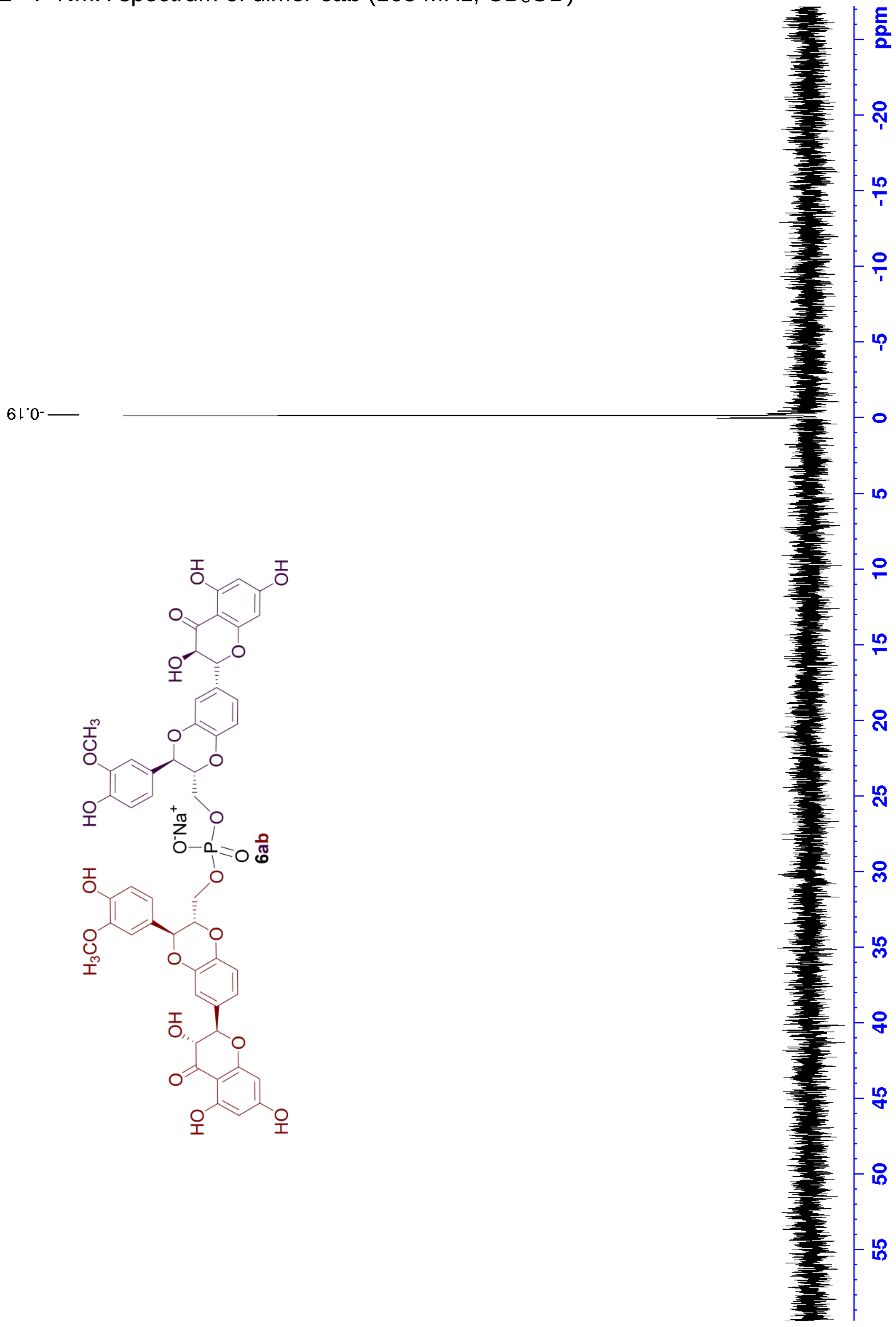

Supplement: Supplementary file 1 [file molecules-27-01702-s001.zip › molecules-1619665-supplementary.pdf]
